# Enterovirus 71 protease 2A^pro^ and 3C^pro^ differentially inhibit the cellular endoplasmic reticulum-associated degradation (ERAD) pathway via distinct mechanisms, and enterovirus 71 hijacks ERAD component p97 to promote its replication

**DOI:** 10.1371/journal.ppat.1006674

**Published:** 2017-10-06

**Authors:** Tao Wang, Bei Wang, He Huang, Chongyang Zhang, Yuanmei Zhu, Bin Pei, Chaofei Cheng, Lei Sun, Jianwei Wang, Qi Jin, Zhendong Zhao

**Affiliations:** 1 MOH Key Laboratory of Systems Biology of Pathogens, Institute of Pathogen Biology, Chinese Academy of Medical Sciences & Peking Union Medical College, Beijing, PR China; 2 Center for Biological Imaging, Institute of Biophysics, Chinese Academy of Sciences, Beijing, PR China; 3 MOH Key Laboratory of Systems Biology of Pathogens and Christophe Mérieux Laboratory, IPB, CAMS-Fondation Mérieux, Institute of Pathogen Biology, Chinese Academy of Medical Sciences & Peking Union Medical College, Beijing, PR China; 4 Center of Clinical Immunology, Chinese Academy of Medical Sciences & Peking Union Medical College, Beijing, PR China; 5 CAMS-Oxford University International Center for Translational Immunology, Chinese Academy of Medical Sciences & Peking Union Medical College, Beijing, PR China; University of Tennessee Health Science Center, UNITED STATES

## Abstract

Endoplasmic reticulum-associated degradation (ERAD) is an important function for cellular homeostasis. The mechanism of how picornavirus infection interferes with ERAD remains unclear. In this study, we demonstrated that enterovirus 71 (EV71) infection significantly inhibits cellular ERAD by targeting multiple key ERAD molecules with its proteases 2A^pro^ and 3C^pro^ using different mechanisms. Ubc6e was identified as the key E2 ubiquitin-conjugating enzyme in EV71 disturbed ERAD. EV71 3C^pro^ cleaves Ubc6e at Q219G, Q260S, and Q273G. EV71 2A^pro^ mainly inhibits the *de novo* synthesis of key ERAD molecules Herp and VIMP at the protein translational level. Herp differentially participates in the degradation of different glycosylated ERAD substrates α-1 antitrypsin Null Hong Kong (NHK) and the C-terminus of sonic hedgehog (SHH-C) via unknown mechanisms. p97 was identified as a host factor in EV71 replication; it redistributed and co-exists with the viral protein and other known replication-related molecules in EV71-induced replication organelles. Electron microscopy and multiple-color confocal assays also showed that EV71-induced membranous vesicles were closely associated with the endoplasmic reticulum (ER), and the ER membrane molecule RTN3 was redistributed to the viral replication complex during EV71 infection. Therefore, we propose that EV71 rearranges ER membranes and hijacks p97 from cellular ERAD to benefit its replication. These findings add to our understanding of how viruses disturb ERAD and provide potential anti-viral targets for EV71 infection.

## Author summary

Understanding of viral-host interactions is important for learning about viral pathogenesis and providing potential anti-viral targets. The protein quality control system, which consists of ERAD and autophagic degradation, is necessary for cellular homeostasis. Our previous studies and others have demonstrated that autophagy is involved in the EV71 lifecycle, but the role of ERAD remains unclear. In this study, we found that EV71 infection also significantly inhibits physiological ERAD at multiple points, causing ERAD substrates to remain tethered in the ER lumen. When exploring the mechanism of EV71-induced ERAD inhibition, data revealed that EV71-encoded viral proteases 2A^pro^ and 3C^pro^ are involved, and they target different molecules with different mechanisms. We also found that ERAD component p97 was essential for the EV71 lifecycle, and it redistributed and co-exists with viral protein and other known replication-related molecules in EV71-induced replication organelles. Thus, we found a novel viral-host interaction that provides new potential anti-viral targets for EV71 infection.

## Introduction

Enterovirus 71 (EV71), which belongs to the *Picornaviridae* family *Enterovirus* genus, is a single-stranded positive-sense RNA virus [1]. This pathogen is the causative agent of hand, foot and mouth disease (HFMD), and is especially the major cause of severe HFMD. Since the first report in the United States in 1974, EV71 outbreaks have been reported around the world, particularly in the Asia-Pacific region in recent years [2]. In China, EV71 caused a severe HFMD outbreak in Fuyang, Anhui province in 2008, and has since become an epidemic problem [3]. The frequency and severity of HFMD have shown an increased annual trend and pose a serious threat to children’s health and social stability in China [4]. However, no effective therapy is currently available for the treatment of this infection and more studies are needed to elucidate the pathogenesis of EV71.

The genome of EV71 encodes eleven proteins, including four viral capsid proteins (VP1–VP4) and seven non-structure proteins (2A–2C, 3A–3D) [1,5]. Among these viral proteins, viral proteases 2A^pro^ and 3C^pro^ have been demonstrated play important roles in virus-host interaction and EV71 pathogenesis. EV71 2A^pro^ has been reported to hijack host cell gene expression by cleaving the eukaryotic initiation factor 4G (eIF4G) and poly(A)-binding protein (PABP) [4,6–8]. It has also been reported to antagonize host innate immunity by down-regulating interferon receptor 1 (IFNAR1) and cleaving mitochondrial antiviral signaling protein (MAVS) [4,9]. EV71 3C^pro^ has been reported to mediate viral immune-evasion by targeting many key components in host innate immunity, including TRIF, IRF7, IRF9, and the RIG-I/IPS-1 complex [5,10–13]. Moreover, 3C^pro^ has also been reported to disrupt host cell gene expression by cleaving CstF-64 [14]. In general, previous studies concerning EV71 viral proteases have focused on innate immunity and gene expression.

In mammalian cells, approximately one-third of the proteins are assembled into mature proteins in the ER [15,16]. This process is tightly monitored by the ER protein quality control (ERQC) system, which is a comprehensive maintenance mechanism for the highly crowded proteins in the ER. This system ensures that only correctly folded and assembled proteins reach their ultimate destination [15,17]. The ERQC system achieves its function via several molecular chaperones and two degradation pathways: the autophagy-lysosome-mediated autophagic degradation and ubiquitin-proteasome-mediated ER-associated degradation (ERAD) pathways [17–20].

Autophagy removes protein aggregates and damaged organelles in double membrane vesicles and degrades them in autolysosomes [21,22]. Several viruses utilize and alter cellular autophagy to facilitate their own replication, including hepatitis C virus (HCV), coronavirus, Dengue virus, influenza A virus, poliovirus (PV), and coxsackievirus B3 (CVB3) [21,23–25]. Huang et al. and our previous studies demonstrated that EV71 can also induce cellular autophagy and exploit autophagy for its own replication [23,26,27]; however, it remains unknown whether ERAD is also affected and involved in EV71 replication.

ERAD is a process that facilitates the degradation of terminally misfolded, misassembled, and metabolically regulated proteins in the ER by retro-translocating them to the cytosol for degradation by the ubiquitin-proteasome system [16,17,28,29]. This process consists of four coupled steps: (i) substrate recognition; (ii) retro-translocation; (iii) ubiquitination; and (iv) 26S proteasome-mediated degradation [16,30,31]. Since ERAD is a key cellular machinery for ensuring correct cell function, it is unsurprising that viruses can manipulate this process for their own benefit. Previous studies demonstrated that different viruses can affect and exploit the ERAD process in different manners [28,31–34]. There are four reasons why viruses exploit ERAD. First, to escape the immune surveillance system by eliminating immune molecules; examples include herpes virus and human immunodeficiency virus (HIV) [28,32]. Second, viruses take advantage of ERAD to achieve the membrane penetration of intact viruses from the ER to the cytosol, such as simian virus 40 (SV40), human BK virus, and murine polymavirus [28,32]. Third, viruses promote ERAD tuning and hijack EDEMosomes to support their replication, including coronaviruses such as severe acute respiratory syndrome coronavirus (SARS-CoV) and mouse hepatitis virus (MHV) [32,35–37]. Finally, viruses can activate ERAD to degrade viral glycoproteins and thereby reduce the viral particle and maintain a chronic infection status; examples include hepatitis B virus (HBV) and hepatitis C virus (HCV) [32,38,39]. However, despite the numerous studies mentioned above, no reports have investigated the relationship between picornaviruses and ERAD.

Here, we demonstrated that EV71 infection inhibits cellular ERAD processes at multiple key ERAD molecules via its proteases 2A^pro^ and 3C^pro^, and ERAD component p97 is involved in EV71 replication. This study reveals a novel relationship between EV71 and cellular ERAD and thus sheds light on the pathogenesis of EV71.

## Results

### EV71 infection inhibited cellular ERAD

ERAD and autophagic degradation are two facets of the host protein quality control system [18]. These biological processes are exploited by various infectious pathogens as survival and proliferation strategies [21,28]. Our previous study also demonstrated that EV71 can take advantage of host autophagy for its own proliferation [27]. However, it remains unknown whether EV71 can affect and modulate the host ERAD machinery. To address this question, we first categorized the ERAD substrates according to their different chaperone systems and established stable cell lines ectopically expressing these substrates. There are two types of ERAD substrate according to their varied chaperone system: calnexin (CNX)/calreticulin (CRT)-dependent substrates and BiP-dependent substrates [16,40,41]. The different types of substrate may be disposed by distinct ERAD sub-pathways and different molecules may be involved.

**Fig 1:**
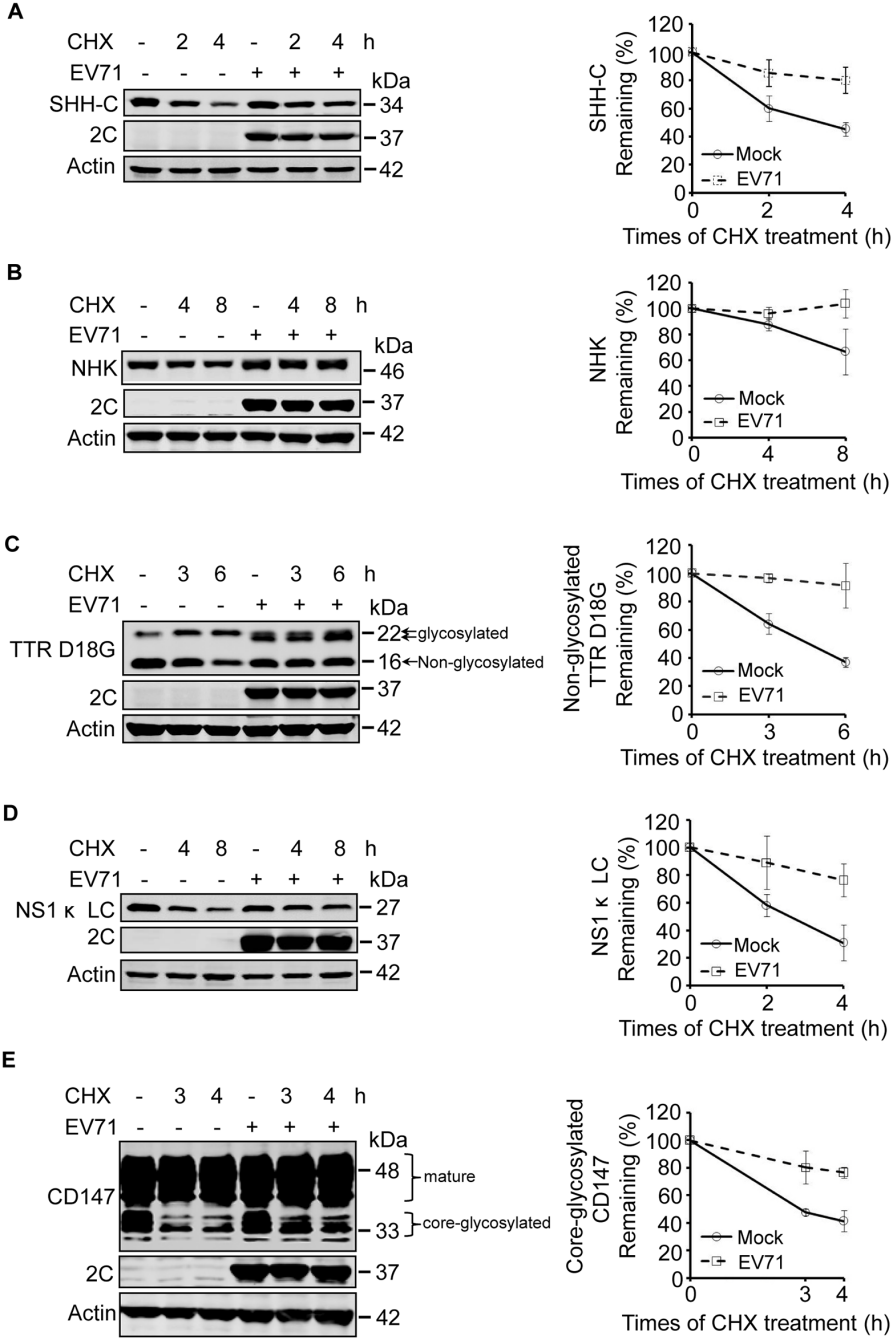
EV71 infection inhibits the degradation of ERAD substrates. (**A–D**) RD cells stably expressing SHH-FLAG (**A**), NHK-FLAG (**B**), TTR D18G-FLAG (**C**), and NS1κ LC-FLAG (**D**) were mock-infected (−) or infected (+) with EV71 (MOI = 10) for different times. Nine hours post-infection the cells were treated with CHX (100 μg/ml) for the indicated times. The cell lysates were separated by SDS-PAGE and then analyzed by western blotting with indicated antibodies to detect the substrates, EV71 2C, and actin; actin was used as the loading control (left panel). The graph shows the quantification of the relative substrate (right panel). The data are presented as means ± SD of three independent experiments. (**E**) RD cells were treated as described in (**A**–**D**), and western blotting was used to detect the expression of CD147, EV71-2C, and actin (left panel). The graph shows the quantification of core-glycosylated CD147 (right panel). The data are presented as means ± SD of three independent experiments.

We first investigated the degradation of two well-characterized CNX/CRT-dependent ERAD substrates: the C-terminus of SHH (SHH-C) and α-1 antitrypsin Null Hong Kong (NHK) [42–47]. Rhabdomyosarcoma (RD) cells stably expressing SHH and NHK were mock infected or infected with EV71 and treated with the protein synthesis inhibitor cycloheximide (CHX) for different times (according to their pre-tested half-life). Western blotting was then used to measure the expression of the substrates. The levels of both SHH-C and NHK were gradually decreased in CHX-treated mock-infected cells in a time-dependent manner. However, the decrease under CHX chase was significantly inhibited in EV71-infected cells (Fig 1A and 1B), indicating that EV71 may inhibit ERAD of SHH-C and NHK.

To further confirm these inhibitory effects, SHH and NHK stable cell lines were treated with tunicamycin (Tun), an inhibitor of N-glycosylation modification, and the fate of already synthesized glycosylated substrates was monitored. In mock-infected cells stably expressing SHH and NHK, treatment with Tun led to a time-dependent decrease in glycosylated SHH-C and NHK, which was accompanied by an increase in the levels of their newly synthesized non-glycosylated forms. However, in EV71 infected cells the degradation of glycosylated SHH-C and NHK was dramatically inhibited (S1A and S1B Fig), confirming the inhibitory effects of EV71 on the ERAD of glycosylated SHH-C and NHK. However, it is worth noting that the *de novo* synthesized non-glycosylated forms of SHH-C and NHK were detectable in mock-infected cells, but not EV71 infected cells (S1A and S1B Fig), indicating that the *de novo* synthesis of SHH and NHK was inhibited by EV71 infection [6–8].

Next, the ERAD of BiP substrates during EV71 infection was evaluated. Transthyretin D18G (the 18th D amino acid mutated to G; TTR D18G) and non-secretory immunoglobulin kappa-type light chain (NS1 κ LC) were selected as representative. TTR is a non-glycosylated soluble secretory protein and TTR D18G is the most destabilized mutant of TTR that is subject to ERAD [48,49]. NS1 κ LC is an unassembled immunoglobulin light chain that is degraded through ERAD [50]. RD cells stably expressing TTR D18G and NS1 κ LC were treated with CHX and their degradation was assessed. The degradation of both substrates was remarkably inhibited in a time-dependent manner during EV71 infection (Fig 1C and 1D), suggesting that EV71 infection also inhibits the ERAD of BiP substrates. It is worth noting that high molecular weight bands of TTR D18G were visible (Fig 1C), which we demonstrated were glycosylated forms of TTR D18G by treating cell lysates with glycosidase PNGase F (S2 Fig). In EV71-infected cells, the molecular weight of glycosylated TTR D18G was decreased, and we speculate that is due to extensive mannose trimming.

Since the above experiments were all performed using stable cell line ectopically expressing different substrates, we next assessed whether EV71 infection could inhibit the degradation of endogenous substrates. Therefore, the degradation of core-glycosylated CD147 (CG), a reported constitutive endogenous substrate was examined during EV71 infection [51]. The CHX chase assay showed that CD147 (CG) was gradually degraded in a time-dependent manner in CHX-treated mock-infected RD cells, However, the degradation was partially inhibited by EV71 infection (Fig 1E), suggesting that EV71 infection also inhibited the ERAD of cellular endogenous substrates.

Considering that EV71 can induce cell apoptosis and that the above experiments were performed with EV71 infection or infection combined with CHX treatment up to 17 h, we checked for cell apoptosis in RD cells under these conditions. The results showed that the proportion of apoptosis was 28.6% in cells infected with EV71 for 17 h, and the combined treatment with CHX for the last 8 h only slightly upregulated the proportion to 32.2% (S1C Fig).

Taken together, the above results demonstrate that EV71 infection inhibits the ERAD of different types of substrates, including both CNX/CRT-dependent glycosylated substrates and BiP-dependent non-glycosylated substrates and cellular constitutively and endogenously expressed substrates.

### EV71 infection caused ERAD substrates to be tethered in the ER

**Fig 2:**
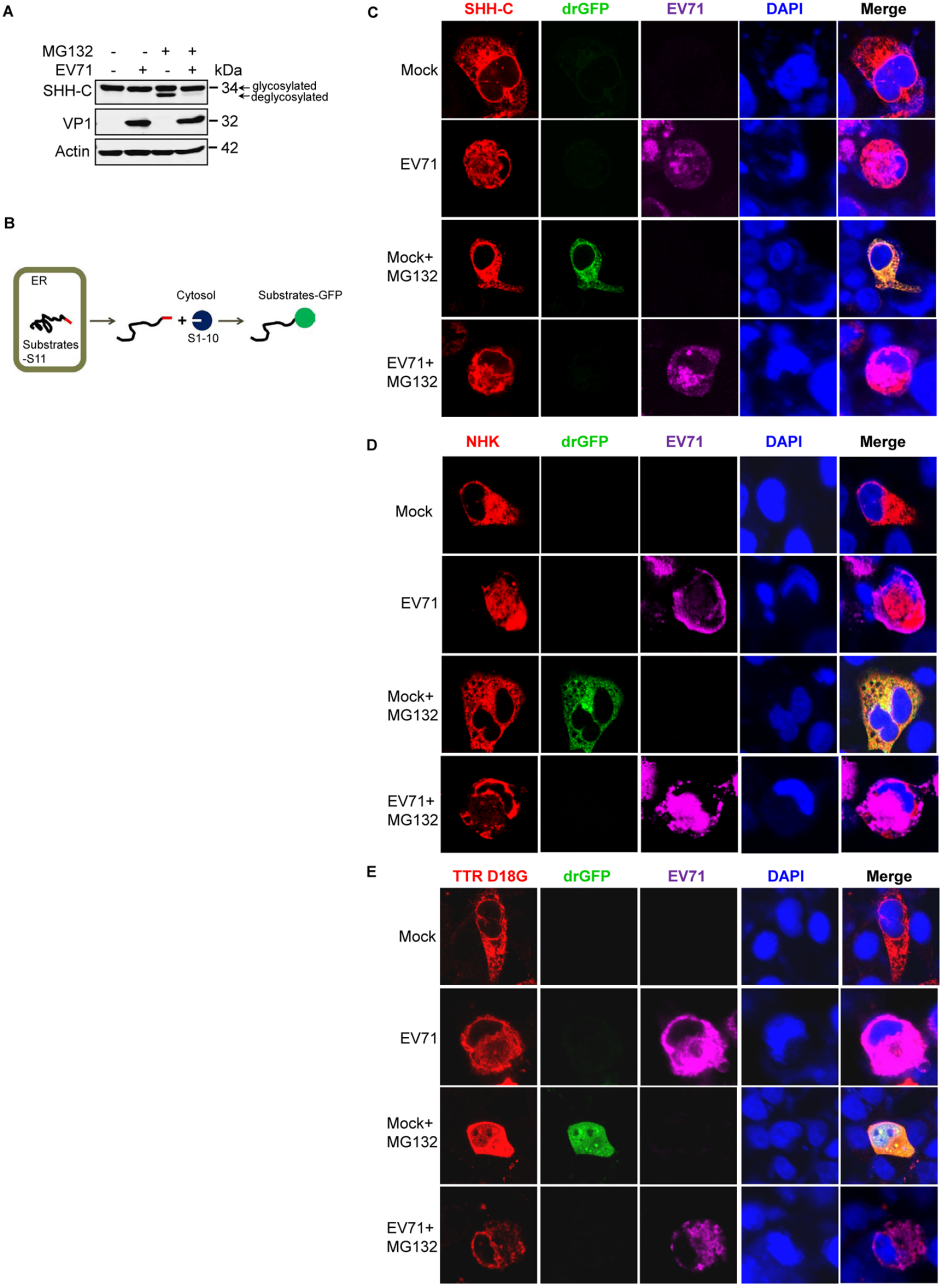
EV71 infection causes ERAD substrates to be tethered in the ER. (**A**) RD cells stably expressing SHH-FLAG were mock-infected (−) or infected (+) with EV71 (MOI = 10) for 7 h and then treated with MG132 (50 μM) for an additional 8 h. Western blotting was then performed with the indicated antibodies. (**B**) Schematic diagram of the dislocation-dependent reconstituted GFP (drGFP) assay. (**C–E**) RD cells were co-transfected with pCMV-SHH-S11-HA (**C**), pCMV-S11-NHK-HA (**D**), or pCMV-TTR D18G-myc-FLAG-S11 (**E**) together with pRRL-S1–10. Twenty-four-hours post-transfection the cells were then mock-infected (−) or infected (+) with EV71 (MOI = 10) for 10 h and then treated with MG132 (50 μM) for another 8 h. Immunostaining was then performed with antibodies against HA (**C** and **D**) or FLAG (**E**) tag and EV71. Fluorescent signals were visualized by laser confocal microscopy (substrates-S11, red; drGFP, green; EV71, purple; nuclei, blue).

Since ERAD is a process that detects misfolded proteins in the ER and extracts them to the cytosol for proteasomal degradation [16,32], we next assessed the location of ERAD substrates in EV71-infected cells. First, SHH-C was used as the substrate. SHH-C is a glycosylated protein that undergoes deglycosylation when retro-translocated to the cytosol. The accumulation of deglycosylated SHH-C when the proteasome is inhibited reflects its retro-translocation degree [43–45]. In cells not treated with the proteasome inhibitor MG132, the deglycosylated SHH-C was not detected in either mock- or EV71-infected cells. However, when cells were treated with MG132, deglycosylated SHH-C was visible as a low-molecular weight band in mock-infected cells, but was barely detectable in EV71-infected cells (Fig 2A). This suggests that the retro-translocation of SHH-C was inhibited by EV71 infection and that SHH-C could be trapped inside the ER lumen.

To further confirm the above conclusion, a previously reported dislocation-dependent reconstituted GFP (drGFP) assay was used to evaluate different substrates, and the principle of this system is illustrated in Fig 2B. Briefly, the GFP molecule is split into two fragments: the C-terminal β-strand (S11) and the remaining 10β strands (S1–10). The S11 is linked to ERAD substrates expressed in the ER lumen, and S1–10 is expressed in the cytosol. When S11-tagged substrates are retro-translocated into the cytosol, S11 is reassembled with S1–10, and the resulting GFP signal is detected when the coupled proteasome degradation is inhibited (Fig 2B) [52]. First, the drGFP assay was used to determine the location of SHH-C in EV71-infected cells. The results showed that in cells not treated with MG132, no GFP signal was observed in either mock-infected or EV71-infected cells. However, the reconstituted GFP signal could be detected in MG132-treated mock-infected cells, but not in EV71-infected cells (Fig 2C). This suggests that EV71 inhibited the retro-translocation activity of SHH-C and that this substrate was trapped inside the ER during infection. The same method was also used to test the retro-translocation activity of NHK and TTR D18G during EV71 infection, and similar results were obtained (Fig 2D and 2E). The drGFP assay was not used to evaluate substrate NS1 κ LC since previous studies reported that NS1 κ LC was retained in the ER lumen and not translocated to the cytosol when cells were treated with a proteasome inhibitor [53]. The above experiments were performed with RD cells expressing different substrates infected with EV71 up to 18 h, and we also used flow cytometry to monitor cell apoptosis under this situation. The results showed that EV71 infection for 18 h caused apoptosis in 30.4% cells, and combined treatment with MG132 in the last 8 h slightly downregulated this proportion (S3 Fig).

Taken together, the above results demonstrated that both CNX-dependent glycosylated substrates and BiP-dependent non-glycosylated substrates were trapped inside the ER during EV71 infection. Therefore, they could not be retro-translocated to the cytosol to undergo subsequent proteasomal degradation.

### EV71 inhibited cellular ERAD pathway at multiple places

**Fig 3:**
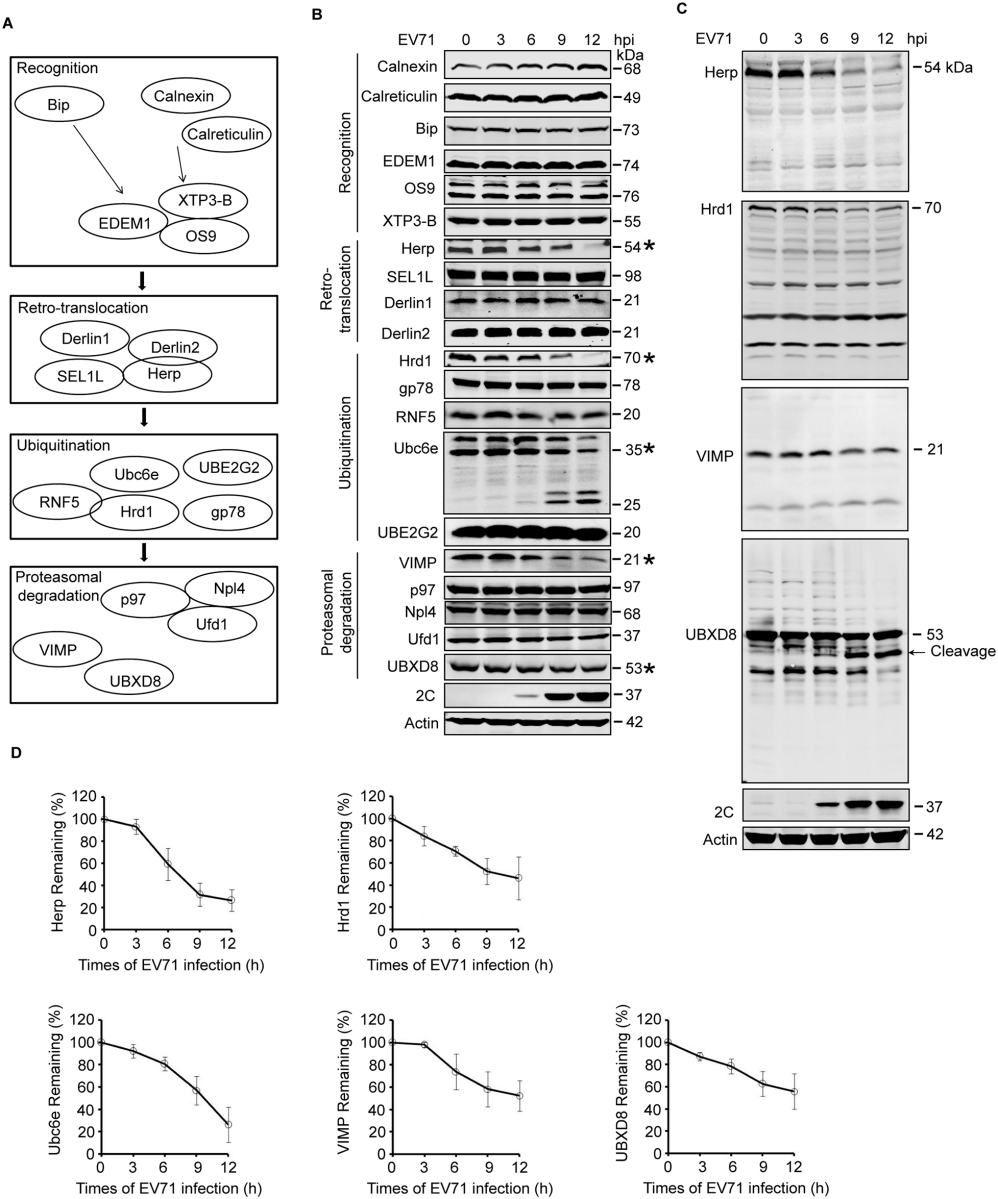
EV71 targets ERAD at multiple points. (**A**) Diagram of the key molecules involved in ERAD. (**B**) RD cells were infected with EV71 (MOI = 10) for the indicated times (hpi: hours post-infection). The cells were then harvested and western blotting was performed using the indicated antibodies to detect the indicated ERAD components, EV71 2C, and actin. The ERAD molecules assessed in this study were separated into four categories: substrate recognition, retrotranslocation, ubiquitination, and proteasomal degradation. Asterisks indicate the molecules that were obviously downregulated. (**C**) Full-size western blots for Herp, Hrd1, VIMP, and UBXD8 described in (**B**). (**D**) Quantification of Ubc6e, Herp, Hrd1, VIMP, and UBXD8 in (**B**). The data are presented as means ± SD of three independent experiments.

The above results demonstrated that EV71 inhibits the degradation of different ERAD substrates. Since different substrates are degraded through distinct sub-pathways, it is likely that EV71 inhibits ERAD at multiple targets or at one shared key point. To clarify the specific molecular mechanisms, ERAD-related molecules were categorized by their different functions and a kinetic study was performed to measure their expression during EV71 infection. The molecules examined were classified into four categories: (1) recognition factors (calnexin, calreticulin, BiP, EDEM1, OS9, and XTP3-B); (2) retro-translocation factors (SEL1L, Herp, Derl1, and Derl2); (3) ubiquitination factors (Hrd1, gp78, RNF5, Ubc6e/UBE2J1, and Ubc7/UBE2G2); and (4) proteasomal degradation factors (VIMP, UBXD8, p97, Ufd1, and Npl4) [16,30,31] (Fig 3A). As expected, EV71 infection downregulated the expression of several molecules in a time-dependent manner, including Herp, Hrd1, Ubc6e, VIMP, and UBXD8 (Fig 3B). Among these, two bands that seemed like cleavage products of Ubc6e could be detected around 25–26 kDa in molecular weight, and their intensity increased as the infection proceeded. Cleavage bands were also detected in the blot of UBXD8, but not in the blots of Herp, Hrd1, and VIMP (Fig 3C).

### EV71 protease 3C^pro^ cleaved Ubc6e at multiple sites

**Fig 4:**
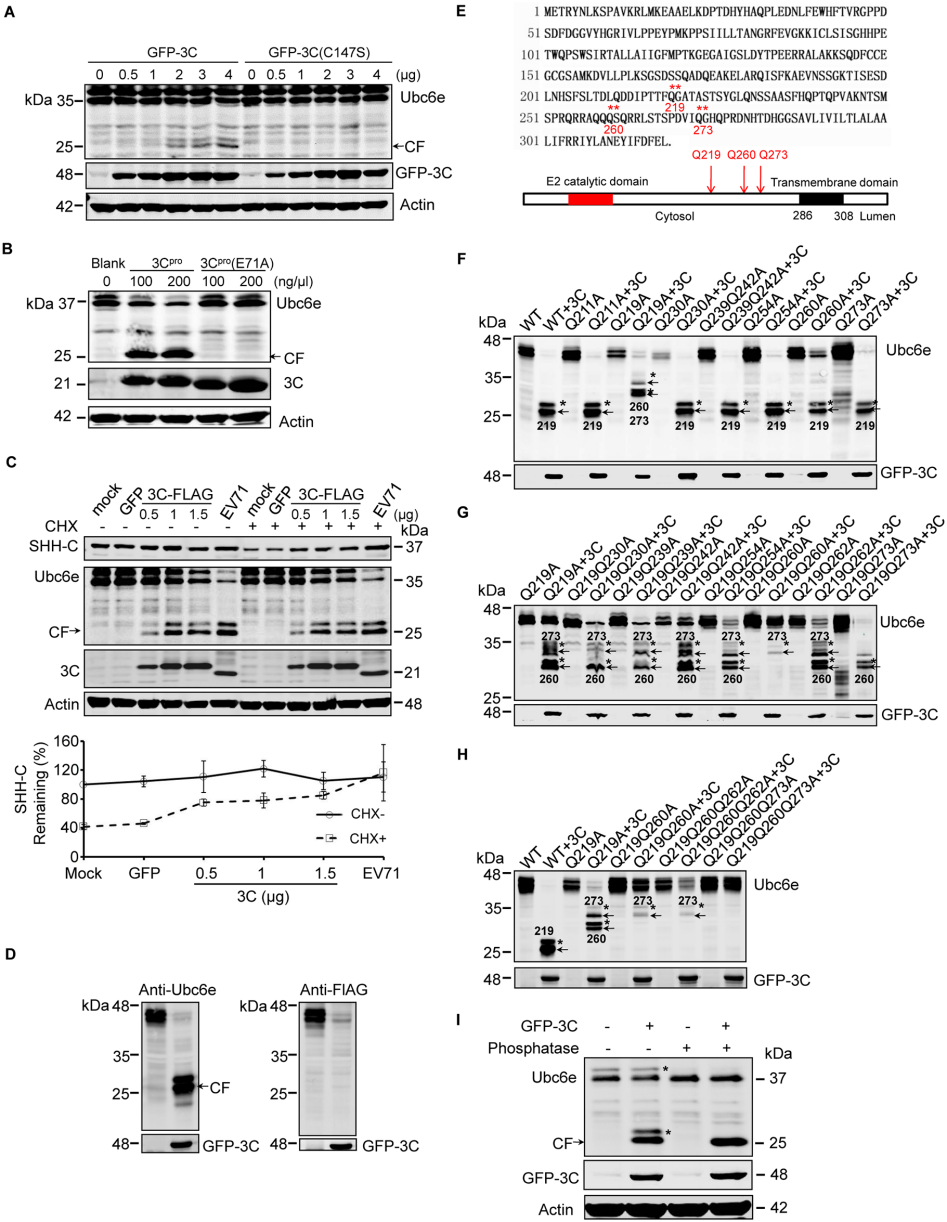
EV71 3C^pro^ cleaves Ubc6e at multiple distinct sites. (**A**) 293T cells were transfected with increasing doses of plasmids (0–4 μg) encoding GFP-3C or GFP-3C(C147S). At 36 h post-transfection, cells lysates were analyzed by western blotting with antibodies against Ubc6e (mouse monoclonal antibody) and GFP. Arrows indicate the cleavage fragments (CF). (**B**) 293T cells lysates were incubated with 100 or 200 ng/μl recombinant 3C^pro^ or 3C protease-dead mutant 3C^pro^(E71A) at 30°C for 2 h, then the mixture was subjected to western blot analysis with indicated antibodies. (**C**) RD cells stably expressing SHH-FLAG were transfected with plasmid encoding GFP or increasing doses of plasmid encoding 3C-FLAG (0–1.5 μg). At 36 h after transfection, the cells were treated with (+) or without (−) CHX for 4 h. Mock- and EV71-infected cells were used as the negative and positive controls for Ubc6e cleavage, respectively. Cells lysates were analyzed by western blotting with antibodies against Flag, Ubc6e, EV71 3C, and Actin. Arrows indicate the cleavage fragments (CF) of Ubc6e. The lower panel graph shows the quantification of SHH-C. The data are presented as means ± SD of two independent experiments. (**D**) 293T cells were transfected with plasmid encoding Ubc6e alone or together with plasmid encoding GFP-3C. At 36 h after transfection, cells lysates were analyzed by western blotting with Ubc6e (left panel) and FLAG (right panel) antibodies. (**E**) Schematic diagram of the Ubc6e amino acid sequence and structural domains (** represents the identified 3C^pro^ cleavage sites on Ubc6e). (**F**–**H**) 293T cells were transfected with plasmids encoding Ubc6e/Ubc6e mutants with or without plasmid encoding GFP-3C. At 36 h after transfection, western blotting was performed to detect Ubc6e cleavage using Ubc6e and GFP antibodies. Arrows indicate cleavage fragments (CF) and asterisks indicate the phosphorylated form of cleavage bands. The numbers on the lane indicate the cleavage sites of cleavage fragments. (**I**) 293T cells were transfected with plasmid encoding Ubc6e alone or together with plasmid encoding GFP-3C. At 36 h after transfection, cells were lysed and cell lysates were incubated with (+) or without (−) Lambda protein phosphatase at 30°C for 30 min, then the lysates were analyzed by western blotting with antibodies against Ubc6e, GFP, and actin.

Previous studies reported that three E2 ubiquitin-conjugating enzymes function in mammalian ERAD: Ubc6e/UBE2J1, UBE2J2, and UBE2G2 [54]. Ubc6e forms an E2-E3 pair with Hrd1 and is considered the principal E2 in cellular ERAD [54–56]. To further clarify the precise mechanism by which Ubc6e is cleaved, we investigated whether EV71-encoded viral proteases 2A^pro^ and 3C^pro^ participate in this process. These viral proteases are responsible for cleaving poly-protein precursors to obtain mature viral proteins, and increasing amounts of evidence have demonstrated that they could cleave various host factors to facilitate viral replication [1,4,5,13]. First, 293T cells were transfected with plasmids encoding EV71 3C^pro^ or a protease-dead mutant of 3C^pro^ (C147S), and Ubc6e cleavage was detected by western blotting. Overexpressed 3C^pro^, but not 3C^pro^(C147S), could cleave Ubc6e in a dose-dependent manner, and the cleavage bands were the same molecular weight as in EV71-infected cells (Fig 4A). This result was also achieved by *in vitro* cleavage assay with recombinant 3C^pro^ and its protease-dead mutant 3C^pro^(E71A) (Fig 4B). We also tested the role of 2A^pro^ in Ubc6e cleavage, and the result showed that no cleavage bands were detected in 2A^pro^-transfected cells (S5 Fig). This suggests that EV71 3C^pro^ cleaves Ubc6e during infection.

Next, we assessed whether 3C^pro^ is the causes of ERAD inhibition. RD cells stably expressing SHH were transfected with increasing doses of plasmids encoding EV71 Flag-tagged 3C^pro^ and western blotting were performed to monitor the degradation of SHH-C under CHX chase. The results showed that SHH-C degradation was inhibited gradually with the increasing expression of EV71 3C^pro^ (Fig 4C). However, the inhibitory effects were not as potent as EV71 infection. This might be due to the limited transfection efficiency of the overexpressed EV71 3C^pro^, or it could be because Ubc6e cleavage is only one of the factors that inhibit ERAD, and other factors could also be involved. Overall, this result suggests that EV71 3C^pro^-induced Ubc6e cleavage could be a crucial mechanism by which EV71 inhibits ERAD.

Then we identified the EV71 3C^pro^ cleavage sites on Ubc6e. 293T cells were co-transfected with plasmids encoding GFP-3C and C-terminal FLAG-tagged Ubc6e, and Ubc6e cleavage was monitored using antibodies against Ubc6e and FLAG (Ubc6e antibody is a mouse monoclonal antibody with unknown epitope). The results showed that the ~25–26 kDa cleavage bands could be recognized by the Ubc6e antibody but not the FLAG antibody (Fig 4D), indicating that these cleavage bands were N-terminal cleavage fragments from Ubc6e. The molecular weight of the cleavage fragments suggested that the cleavage sites might be located at the region of amino acids 200–300 of Ubc6e (Fig 4E). Since picornavirus 3C^pro^ preferentially cleaves glutamine-glycine (Q-G), glutamine-alanine (Q-A), and glutamine-serine (Q-S) bonds in viral polyproteins and cellular targets [11,57], a panel of site-directed mutants with Q mutated to A within the 200–300 amino acid (aa) region was constructed. Then, 293T cells were co-transfected with the GFP-3C^pro^ and Ubc6e/Ubc6e mutants to map the cleavage sites. As illustrated in Fig 4F, all the mutants could be cleaved by 3C^pro^ as before. However, the cleavage bands from mutant Q219A were shifted to a molecular weight of ~30 kDa (Fig 4F). This indicates that the 219^th^ glutamine of Ubc6e (Q219) is one of the cleavage sites and that another cleavage site(s) exists that is located closer to the C-terminus of Ubc6e. To further identify the remaining cleavage sites, a new panel of double-site mutants (Q to A) was constructed within the 219–300 aa region based on the Q219A mutant. These series mutants revealed that all the double-site mutants could be cleaved by 3C^pro^; however, the cleavage bands were changed in cells transfected with mutant Q219Q260A and Q219Q273A. This indicates that both Q260 and Q273 are the cleavage sites of 3C^pro^ on Ubc6e (Fig 4G). To further confirm this result, a third round of screening was conducted using triple-site Q to A mutants that were generated based on Q219Q260A including Q219Q260Q262A and Q219Q260Q273A. The results showed that triple-site mutant Q219Q260Q273A was totally resistant to 3C^pro^ cleavage; thus, Q260 and Q273 were identified as the second and third cleavage sites (Fig 4H). Taken together, the above results demonstrate that EV71 3C^pro^ cleaves Ubc6e at Q219G, Q260S, and Q273G. These sites are all located on the cytoplasmic side of Ubc6e, and cleavage at these points will lead to the release of the E2 catalytic domain of Ubc6e into the cytosol and inactive Ubc6e at the ER membranes. When all the cleavage sites were identified, we re-analyzed the results of Fig 4E–4G and speculated that each cleavage fragment was accompanied by a phosphorylated form with a lower migrating speed [58,59]. We also confirmed this by treating cell lysates with phosphatase and detected the cleavage of Ubc6e; the results showed that both the dual-band of Ubc6e and cleaved Ube6e changed to a single-band when treated with phosphatase (Fig 4I).

### EV71 protease 2A^pro^ was responsible for the decreased expression of Herp and VIMP

**Fig 5:**
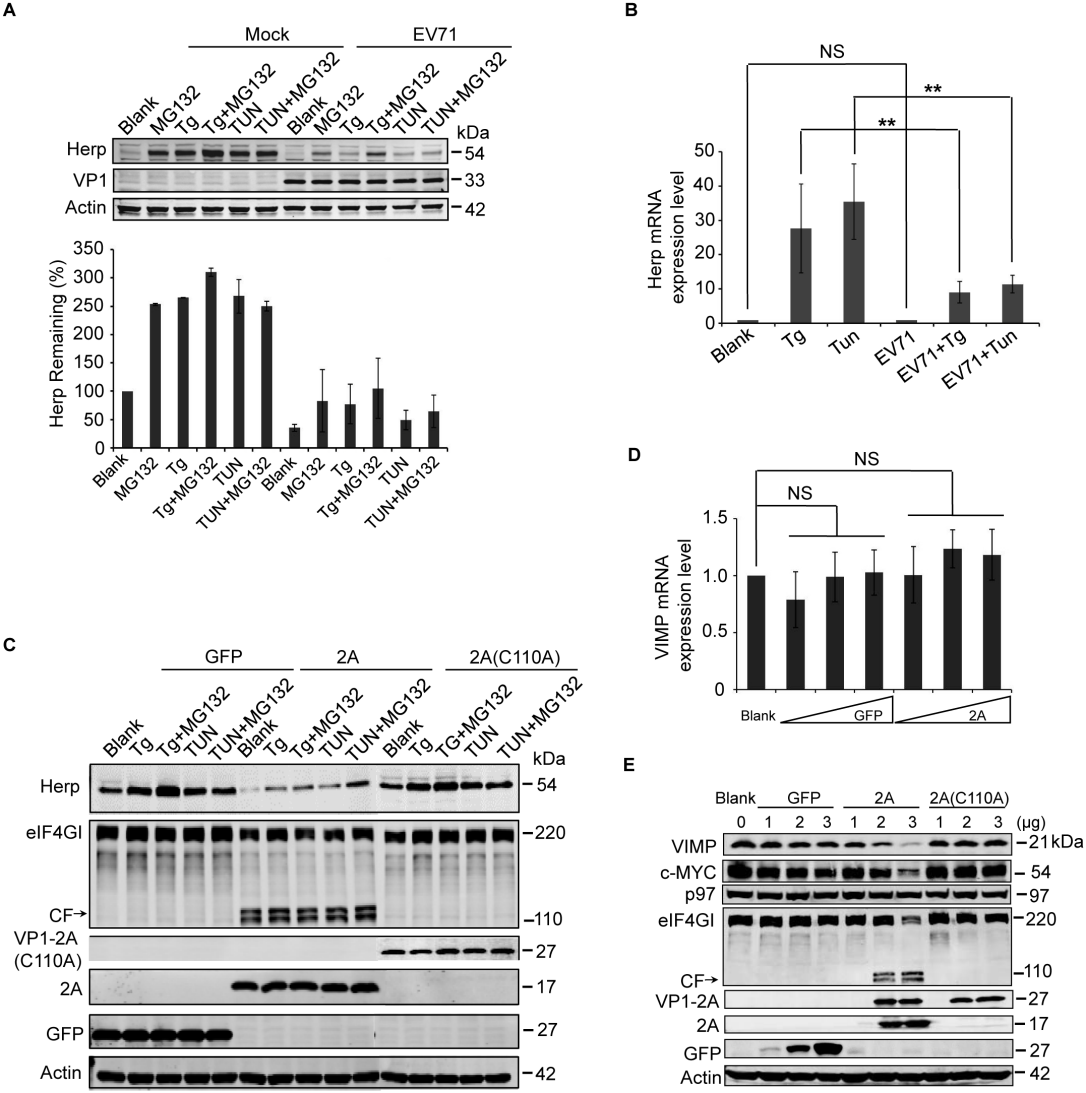
EV71 2A^pro^ inhibits the biosynthesis of Herp and VIMP. (**A**) RD cells were mock-infected (−) or infected (+) with EV71 (MOI = 10) for 9 h and then treated with MG132 (50 μM), Tg (300 nM), Tg plus MG132, Tun (10 μg/ml), Tun plus MG132 for an additional 6 h. Then, the cells were harvested and analyzed by western blotting with antibodies against Herp, EV71 VP1, and actin. The lower panel graph shows the quantification of Herp. The data are presented as means ± SD of two independent experiments. (**B**) RD cells were mock-infected (−) or infected (+) with EV71 (MOI = 10) for 9 h and then treated with Tg (300 nM) or Tun (10 μg/ml) for 6 h to induce Herp expression. Then, the mRNA expression levels of *Herp* were evaluated by quantitative real-time PCR. The data are presented as means ± SD of two independent experiments. NS, non-significant, P≥0.05; ** P<0.01. (**C**) BSRT7 cells were transfected with pcDNA3.1-EGFP, pcDNA3.1-IRES-2A, or pcDNA3.1-IRES-2A(C110A). At 36 h post-transfection, cells were treated with Tg (300 nM), Tg plus MG132 (50 μM), Tun (10 μg/ml), or Tun plus MG132 for 6 h. Then, cell lysates were analyzed by western blotting with antibodies against Herp, eIF4GI, V5, GFP, and actin. eIF4GI was included as a positive control of protease activity of 2A^pro^, and arrows indicate the cleavage fragments (CF) of eIF4GI. (**D**) BSRT7 cells were transfected with increasing doses of pcDNA3.1-EGFP plasmid or pcDNA3.1-IRES-2A plasmid (1–3 μg). At 36 h post-transfection, the cells were harvested, RNA was extracted, and quantitative real-time PCR was used to analyze *VIMP* mRNA expression. The data are presented as means ± SD of three independent experiments. NS, non-significant, P≥0.05. (**E**) BSRT7 cells were transfected with increasing doses of pcDNA3.1-EGFP, pcDNA3.1-IRES-2A, or pcDNA3.1-IRES-2A(C110A) plasmids (1–3 μg). At 36 h post-transfection, the cells were harvested and cell lysates were analyzed by western blotting with antibodies against VIMP, c-MYC, p97, eIF4GI, GFP, V5, and actin.

Next, the mechanism behind the decreased expression of Herp and VIMP was examined. Herp, whose expression is strongly induced by the unfolded protein response (UPR), is involved in the turnover of ERAD substrates [60–62]. It participates in building the dislocation machinery around Hrd1 and itself has a very fast turnover [44,63–65]. To further explore the reason behind the Herp reduction, we first assessed whether the reduction of Herp was caused by accelerated degradation or blocked synthesis. Mock- and EV71-infected RD cells were treated with the ER stress inducers thapsigargin (Tg) and tunicamycin (Tun) to induce Herp expression, and the degree of upregulation was compared between mock- and EV71-infected cells. Tg and Tun could induce Herp expression efficiently in mock-infected cells, especially when the cells were treated with ER stress inducers combined with the proteasome inhibitor MG132. However, the same treatment induced less Herp expression in EV71-infected cells, implying that EV71 infection may inhibit the *de novo* synthesis of Herp and that Herp is degraded via proteasomal degradation (Fig 5A). Cell apoptosis in EV71-infected cells and infected cells combined with different chemical treatments as shown in Fig 5A were also checked, and no obvious differences were observed between different groups (S6 Fig).

Since EV71 can inhibit host gene expression at the protein translation level [4], we next assessed whether the EV71-induced Herp reduction was caused by blocked protein translation. To test this hypothesis, the impact of EV71 on mRNA transcription was checked. Mock- and EV71-infected RD cells were treated with Tg and Tun, and *Herp* mRNA expression was assessed using real-time PCR. Tg and Tun induced *Herp* expression efficiently in mock-infected cells, but the extent of upregulation was reduced by 2/3 in EV71-infected cells. However, there were no differences of basal *Herp* expression between mock- and EV71-infected cells (Fig 5B). This suggests that EV71 might inhibit basal Herp synthesis at the translational level and inhibit Tg- and Tun-induced Herp synthesis at both the transcriptional and translational levels.

EV71 2A^pro^ can inhibit protein translation in host cells by cleaving many host factors related to the translation process, including eukaryotic translation initiation factor (eIF4GI), eIF4GII, and poly-A binding protein (PABP) [4,6–8]. Therefore, we assessed whether EV71 2A^pro^ blocked the protein synthesis of Herp in a previously reported BSRT7 cell line that stably expresses T7 RNA polymerase and effectively avoids the difficulties in expressing 2A^pro^ in eukaryotic cells [4,8,66]. Using GFP and 2A protease-dead mutant 2A(C110A) as the control, Tg and Tun induced Herp expression to a much weaker extent in 2A-transfected cells compared with control cells (Fig 5C), suggesting that EV71 2A^pro^ could inhibit Herp synthesis.

Next, the mechanism of VIMP reduction during EV71 infection was investigated. VIMP is a valosin-containing protein (VCP)-interacting membrane protein [65]. It is an important component of the ERAD complex because it can recruit p97 and other cofactors to the ER membrane for retro-translocation [67–69]. A CHX chase assay revealed that VIMP is a short-lived protein, similar to Herp (S4 Fig). Therefore, it is possible that EV71 inhibits VIMP and Herp expression via a similar mechanism. To confirm this, the BSRT7 cell line was transfected with EV71 2A, and VIMP expression was monitored at both the mRNA and protein levels. The results showed that there were no differences of *VIMP* mRNA expression between GFP and 2A-transfected cells (Fig 5D). However, the protein expression of VIMP was significantly downregulated in 2A-transfected cells, but not GFP and 2A(C110A)-transfected cells at the protein level, in a dose-dependent manner (Fig 5E), suggesting that the EV71 2A^pro^ also inhibits the protein synthesis of VIMP.

Taken together, the above results demonstrate that EV71 2A^pro^ inhibits protein synthesis of Herp and VIMP. Although 2A^pro^ of picornaviruses is well known to inhibit cap-dependent host cell translation by cleaving several host factors related to the translation process [4,5,7,8], we thought that its effect on host cell protein expression mainly embodied on short-lived proteins, like Herp and VIMP in this study. We also included c-MYC, another reported short-lived protein [70,71], and p97, which is not short-lived, as controls and detected their expression in 2A- and 2A(C110A)-transfected cells. As expected, the expression of c-MYC but not p97 was downregulated in 2A-transfected cells (Fig 5E).

### Herp and Ubc6e differentially participate in the ERAD of different substrates

**Fig 6:**
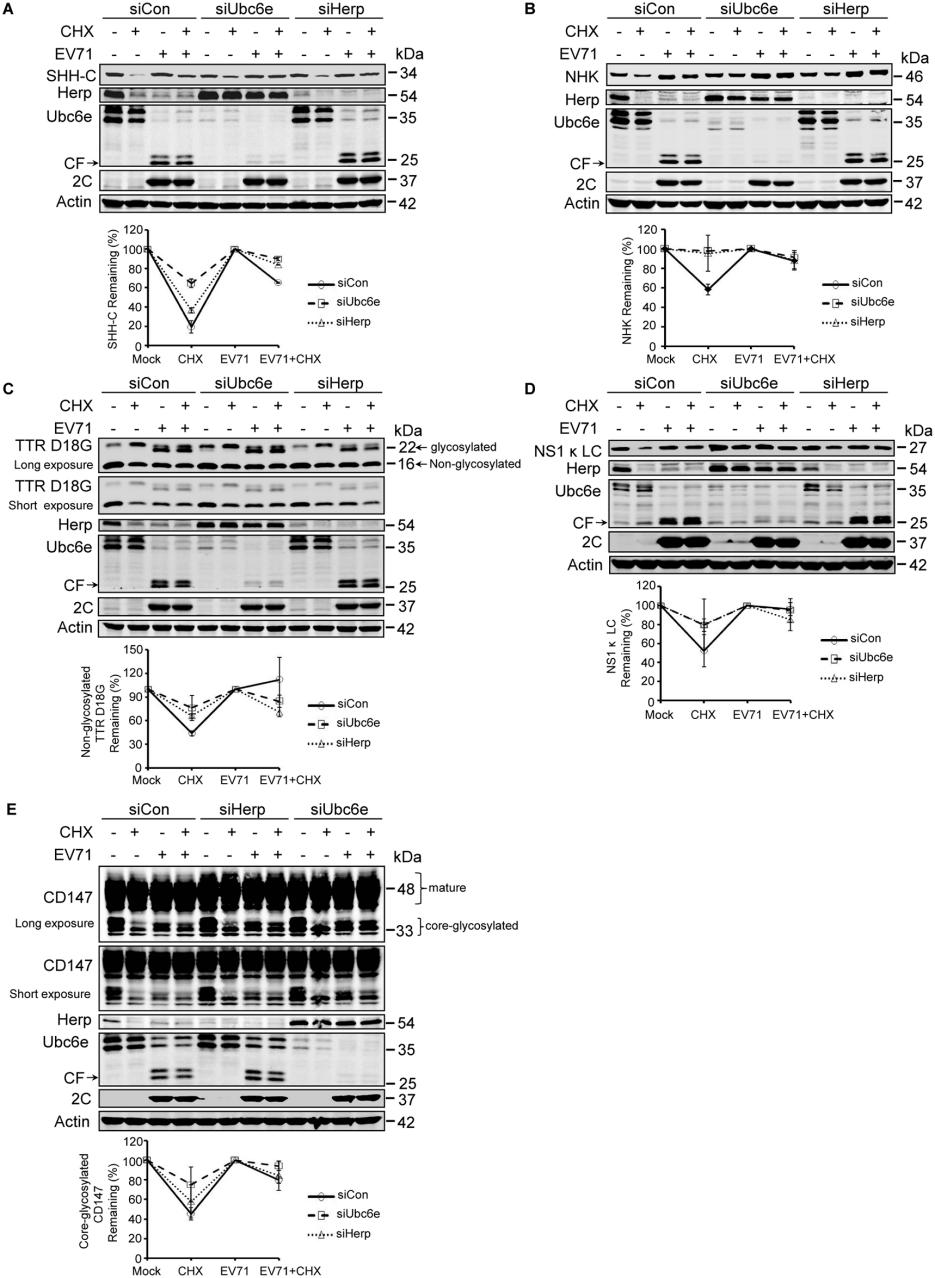
Various substrates rely differently on Herp and Ubc6e for degradation. (**A**–**D**) RD cells stably expressing SHH-FLAG (**A**), NHK-FLAG (**B**), TTR D18G-FLAG (**C**), and NS1κLC-FLAG (**D**) were transfected with control siRNA and siRNA targeting Ubc6e or Herp. At 36 h post-transfection, cells were mock infected (−) or infected (+) with EV71 (MOI = 10) for 12 h and then treated with (+) or without (−) CHX for an additional 4 h. The cells were then harvested and cell lysates were analyzed by western blotting with antibodies against FLAG, Herp, Ubc6e, 2C, and actin. (**E**) RD cells were treated as described in (**A–D**) and western blotting was performed using antibodies against CD147, Herp, Ubc6e, 2C, and actin. The graphs show the quantification of the relative substrate (lower panel). The data are presented as means ± SD of two independent experiments.

We next evaluated of Herp and Ubc6e in the degradation of different ERAD substrates. First, their role in the degradation of SHH-C was examined. RD cells stably expressing SHH were transfected with siRNA to silence Ube6e and Herp. The cells were then chased with CHX under mock- or EV71-infection conditions and the degradation of SHH-C was assessed by western blotting. Knocking down Ubc6e but not Herp significantly inhibited the degradation of SHH-C under CHX chase in RD cells (Fig 6A), suggesting that the ERAD of SHH-C was Ubc6e-dependent and Herp-independent. It is worth noting that silencing Ubc6e substantially upregulated the expression of Herp, suggesting that Ubc6e is a critical modulator of Herp degradation. Next, the same method was used to evaluate the role of Ubc6e and Herp in NHK degradation. The results differed to those of SHH-C, since knocking down either Ubc6e or Herp inhibited the degradation of NHK (Fig 6B). This suggests that both Ubc6e and Herp are required for its degradation. Moreover, since the E2 responsible for NHK ERAD has not yet been identified, this study strongly suggests that Ubc6e is also the E2 for NHK degradation.

Next, the contribution of Ubc6e and Herp to the ERAD of TTR D18G and NS1κ LC was validated. The depletion of Ubc6e significantly inhibited the degradation of non-glycosylated TTR D18G and upregulated the basal intracellular expression of glycosylated TTR D18G (Fig 6C), suggesting that Ubc6e is a key element for the degradation of TTR D18G. Since knocking down Herp also inhibited the degradation of non-glycosylated TTR D18G (Fig 6C), these data suggest that Herp is also involved in the ERAD of non-glycosylated TTR D18G. Regarding NS1κ LC, the results showed that knocking down either Ubc6e or Herp inhibited the degradation of NS1κ LC in CHX-chased cells and obviously upregulated the basal expression of NS1κ LC (Fig 6D). This suggests that both Ube6e and Herp were also essential to the ERAD of NS1κ LC.

Finally, we determined whether the degradation of the endogenous substrate CD147 (CG) requires Ubc6e and Herp. Similar experiments as those described above were performed. As illustrated in Fig 6E, knocking down either Ubc6e or Herp attenuated the degradation of CD147 (CG), suggesting that Ubc6e and Herp were also involved in the ERAD of the endogenous ERAD substrate CD147 (CG).

Taken together, these data demonstrate that Ubc6e functions as a key E2 to play a role in the ERAD of all the substrates tested in this study. In addition, Herp is required for the ERAD degradation of NHK, TTR D18G, NS1κ LC, and endogenous CD147, but not SHH-C. Herp differentially participates in the degradation of different glycosylated ERAD substrates SHH-C and NHK, suggests that factors other than the binding chaperone and glycosylation determine the substrate specificity of Herp.

#### The ERAD component p97 and its ATPase activity are essential for EV71 replication, and it is co-localized with EV71 2C protein

**Fig 7:**
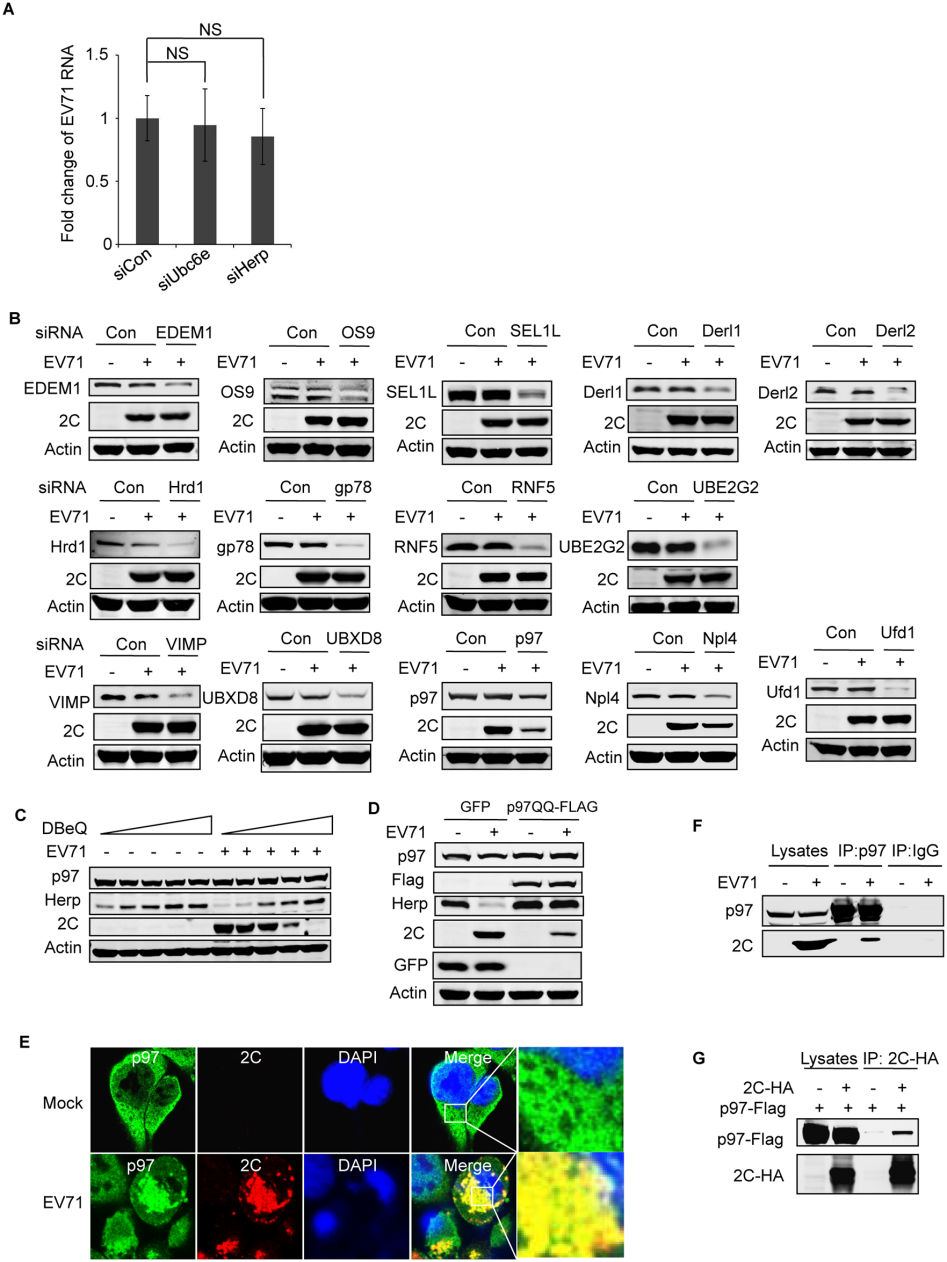
p97 and its ATPase activity are essential for EV71 replication, and it co-localizes with EV71 2C. (**A**) RD cells were transfected with control siRNA or siRNA targeting Ubc6e or Herp. At 36 h post-transfection, cells were infected with EV71 (MOI = 10) for 12 h. Then real-time PCR was used to qualified viral RNA replication. Data are expressed as fold-change of the EV71 RNA level relative to cells transfected with siRNA control. The data were examined in at least two independent experiments; NS, non-significant, P≥0.05. (**B**) RD cells were transfected with control siRNA and siRNA targeting different ERAD components including EDEM1, OS9, SEL1L, Derl1, Derl2, Hrd1, gp78, RNF5, UBE2G2, VIMP, UBXD8, p97, Npl4, and Ufd1. At 36 h post-transfection, cells were mock infected (−) or infected (+) with EV71 (MOI = 10) for 12 h. Then, western blotting was carried out to detect the knockdown efficiency of different molecules, EV71 2C, and actin. (**C**) RD cells were pre-treated with increasing doses of DBeQ (0–10 μM) for 3 h and then mock-infected (−) or infected (+) with EV71 (MOI = 10) for 12 h. The cell lysates were then analyzed by western blotting with the indicated antibodies. Herp expression was used as an indicator of DBeQ efficiency, and actin was used as the loading control. (**D**) RD cells were transfected with plasmids encoding GFP or p97QQ-FLAG (dominant negative p97 mutant). At 36 h after transfection, the cells were mock-infected (−) or infected (+) with EV71 (MOI = 10) for 12 h, and the cell lysates were analyzed by western blotting with the indicated antibodies. (**E**) RD cells were mock-infected or infected with EV71 (MOI = 10) for 12 h, and immunostaining was then performed to detect the intracellular distribution of p97 and EV71 2C (p97, green; 2C, red; nuclei, blue). Insets show magnified views of the merged channels in the boxed region. (**F**) RD cells were mock infected (−) or infected (+) with EV71 (MOI = 10) for 12 h, and cell lysates were immunoprecipitated (IP) with p97 mouse monoclonal antibody or control mouse IgG. Cell lysates and precipitates were analyzed by western blotting with antibodies against p97 and EV71 2C. (**G**) 293T cells were cotransfected with empty vector (control) or plasmids encoding 2C-HA and p97-FLAG. At 36 h after transfection, cell lysates were immunoprecipitated with HA antibodies. The cell lysates and precipitates were then analyzed by western blotting with antibodies against p97-FLAG and 2C-HA.

To clarify the significance of EV71 inhibiting cellular ERAD, we first examined whether key ERAD components could impact the lifecycle of EV71. Although Ubc6e and Herp are viral targets of cellular ERAD, our above results showed their knockdown did not change viral protein expression in infected cells (Fig 6A–6E). So we further checked the viral RNA in Ubc6e or Herp knockdown cells, and the result showed that viral RNA replication was not affected by their knockdown (Fig 7A). Then we used siRNA to silence different ERAD components besides of Ubc6e and Herp and checked their role in EV71 replication. The results showed that the depletion of ATPase p97 remarkably inhibited EV71 replication, and the depletion of p97 cofactor Npl4 had a slight inhibitory effect (Fig 7B). This suggests that the p97 complex, and especially p97 itself, is a critical host factor that is required for EV71 replication. To further explore whether the ATPase activity of p97 is required for EV71 replication, N2, N4-dibenzylquinazoline-2, 4-diamine (DBeQ), a reversible small molecule inhibitor, and p97 dominant negative mutant (p97QQ) [52,72], were used. Pretreating RD cells with DBeQ and transfecting RD cells with p97QQ remarkably attenuated EV71 infection in a dose-dependent manner (Fig 7C and 7D), suggesting that ATPase activity is required for EV71 replication.

According to the above results, we hypothesized that p97 may be hijacked by EV71 from the disabled ERAD pathway to promote its own replication. To test this hypothesis, we examined whether the distribution of p97 is changed by multicolor fluorescent confocal microscopy. The distribution of p97 had changed significantly in EV71-infected cells; the original evenly distributed pattern had changed to a non-uniform state, and most p97 accumulated around the wrinkled nuclei. In addition, p97 co-localized with EV71 2C (Fig 7E), suggesting that p97 and 2C jointly participate in EV71 replication. Next, endogenous co-immunoprecipitation experiments were performed in EV71-infected cells with p97 antibody, the results showed 2C immunoprecipitated by p97 antibody but not control IgG (Fig 7F), indicating p97 and EV71 2C protein may interact in infected cells. Since 2BC and 3AB protein of poliovirus were also reported interact with p97 [73], the interaction of p97 and 2C we observed in EV71 infected cells may be indirectly and mediated by other viral proteins. To test this possibility, exogenous co-immunoprecipitation experiments were further performed in 293T cells co-transfected with plasmids encoding HA-tagged EV71 2C protein and Flag-tagged p97 protein. The results showed that p97 also co-immunoprecipitated with 2C (Fig 7G), indicating p97 and 2C could interact with each other with no aid of other viral proteins. However, these results cannot exclude the possibility that other viral proteins also interact with p97.

Taken together, these results strongly suggest that p97 co-exists with EV71 2C and might other viral proteins and is involved in EV71 replication. Furthermore, we proposed that the site of aggregation of p97 and EV71 viral proteins in the infected cells might be the site of viral replication.

#### p97 and EV71 2C co-localized in viral replication organelles (ROs) during EV71 infection

**Fig 8:**
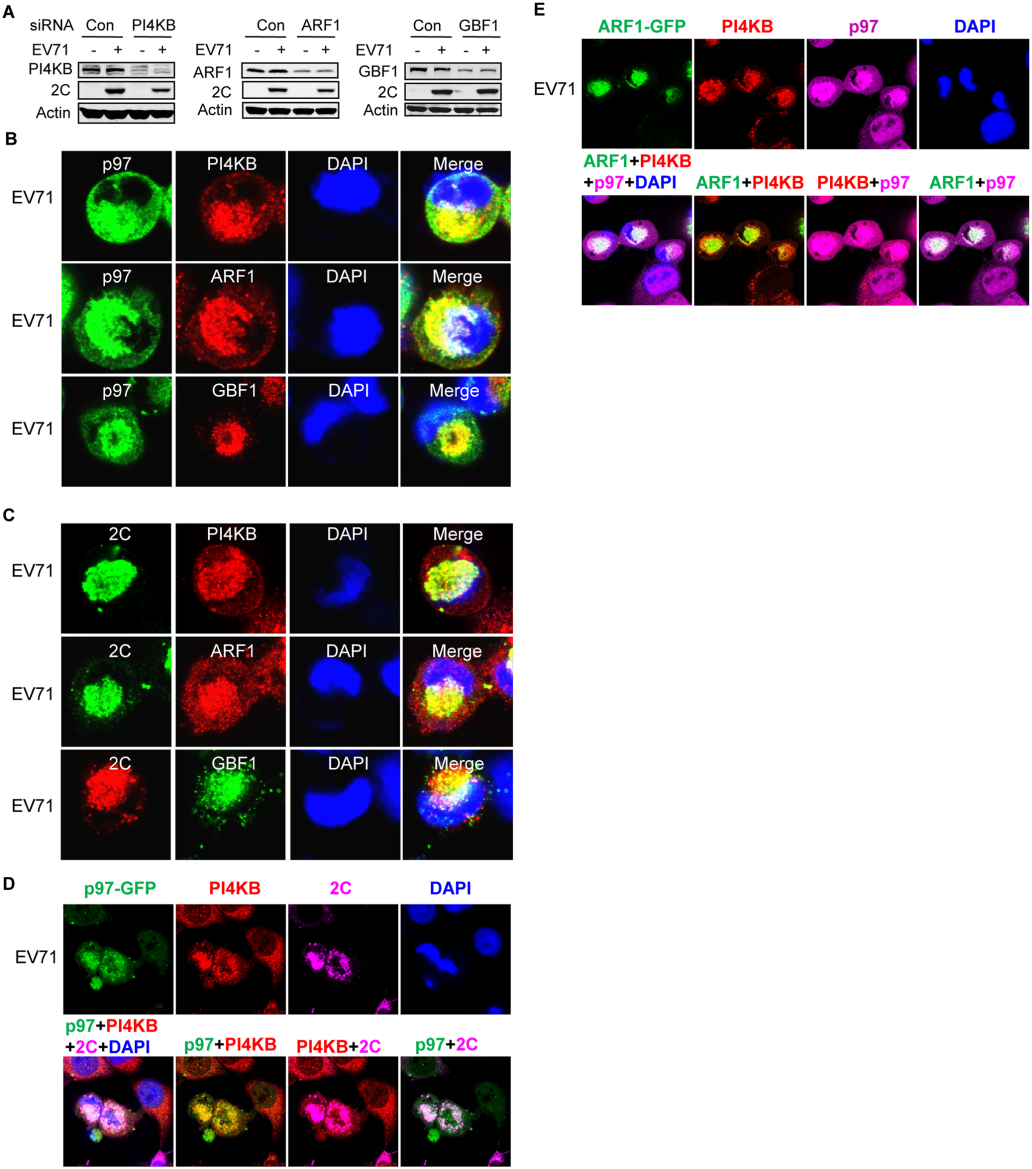
p97 and 2C co-localize with PI4KB, ARF1, and GBF1 on viral replication organelles. (**A**) RD cells were transfected with control siRNA or siRNAs targeting PI4KB, ARF1, and GBF1. At 36 h post-transfection, cells were mock-infected (−) or infected (+) with EV71 (MOI = 10) for 12 h. The cell lysates were then analyzed by western blotting with the indicated antibodies. (**B**) RD cells were infected with EV71 (MOI = 10) for 12 h; the cells were then fixed and stained with mouse anti-p97 and rabbit anti-PI4KB/ARF1/GBF1 antibodies (nuclei, blue; p97, green; PI4KB/ARF1/GBF1, red). (**C**) RD cells were treated as described in (**B**) and then stained with mouse anti-2C and rabbit anti-PI4KB/ARF1/GBF1 antibodies. (**D**) RD cells stably expressing p97-GFP were infected EV71 (MOI = 10) for 12 h, fixed, and stained with rabbit anti-PI4KB and mouse anti-2C antibodies (nuclei, blue; p97, green; PI4KB, red; 2C, purple). The upper panels show an image of each channel and the lower panels show merged pictures of two or four channels as indicated. (**E**) Cells were treated as described in (**D**), except that RD cells were stably expressing ARF1-GFP and stained with rabbit anti-PI4KB and mouse anti-p97 antibodies (nuclei, blue; ARF1, green; PI4KB, red; p97, purple).

Many RNA viruses rearrange intracellular membranes to generate replication organelles (ROs) [74,75]. The ROs of picornaviruses have been reported to formed at ER-Golgi interface, and utilize host factors including phosphatidylinositol 4-kinase IIIβ (PI4KB), ADP-ribosylation factor 1 (ARF1), and Golgi brefeldin A-resistant guanine nucleotide exchange factor 1 (GBF1) [74,76–79]. To test whether similar ROs also formed in EV71-infected cells, we first checked whether the above-mentioned host factors are also involved in the lifecycle of EV71 by siRNA knock-down. The results showed that knocking down either PI4KB or ARF1, but not GBF1, inhibited EV71 propagation (Fig 8A), suggesting that EV71 induced similar but not exactly the same ROs as other picornaviruses. Next, immunofluorescence staining was performed to assess whether the intracellular distributions of PI4KB, ARF1, and GBF1 had changed in infected cells and whether p97 and EV71 2C co-localized with these factors. PI4KB, ARF1, and GBF1 all redistributed to the perinuclear region of infected cells and co-localized with p97 and 2C (Fig 8B and 8C). To further demonstrate that the site of co-localization with the host factors p97 and 2C are the ROs of infected cells, co-localization assays were performed between PI4KB-p97-2C and PI4KB-ARF1-p97. All three molecules co-localized in the perinuclear region (Fig 8D and 8E), suggesting that the aggregation foci of p97 and 2C in the perinuclear region were actually viral ROs.

#### EV71 hijacks p97 and utilizes ER-derived membranes to form viral ROs to support its replication

**Fig 9:**
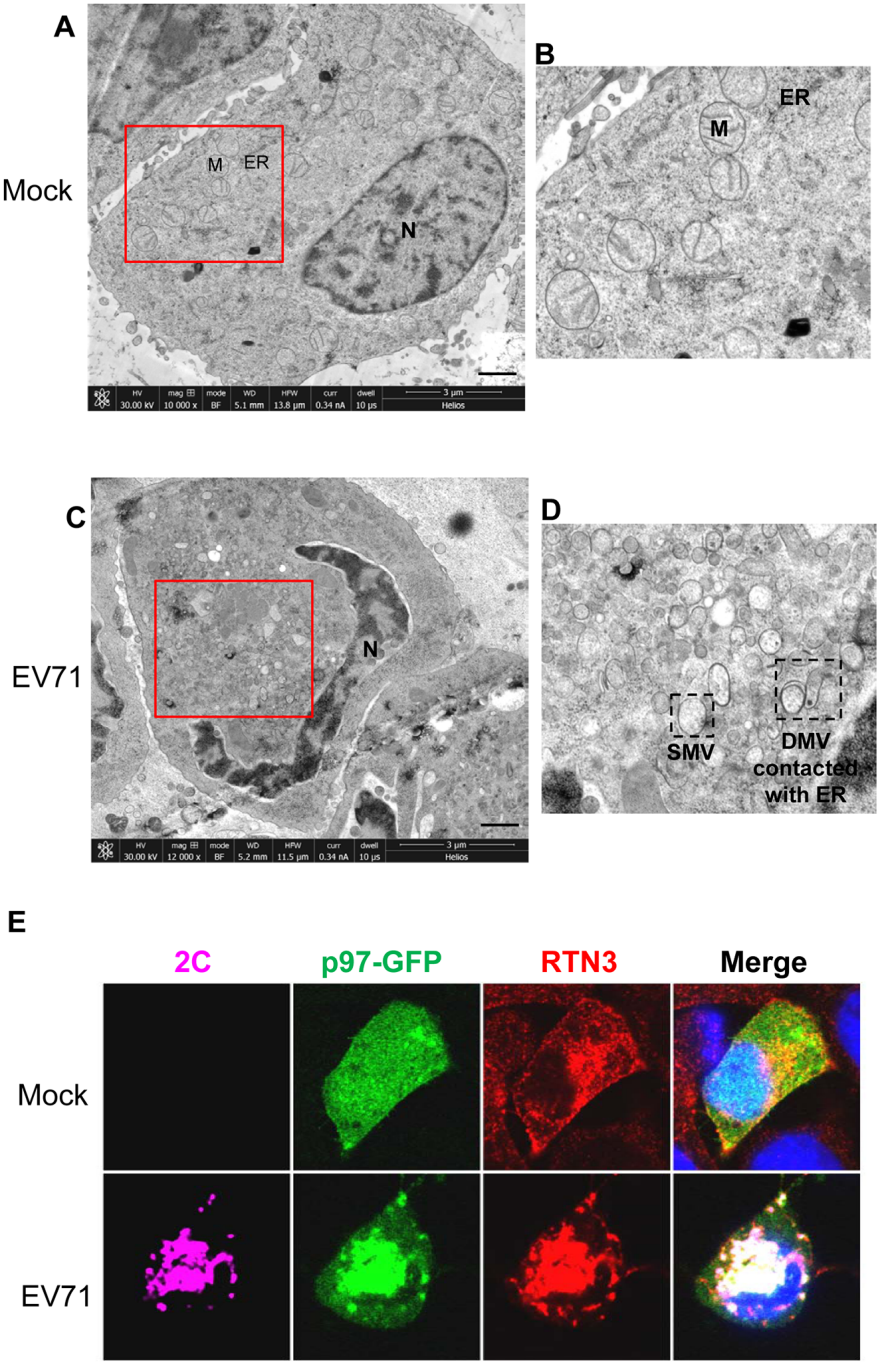
EV71 induces ROs in infected cells and redistributes RTN3 to viral ROs during infection. (**A**) Low-magnification electron micrographs of mock infected RD cells. N, nucleus; M, mitochondria; ER, endoplasmic reticulum. Scale bar, 1 μm. (**B**) Magnified view of the boxed region in (**A**). (**C**) Low-magnification electron micrographs of EV71-infected RD cells (MOI = 10, 12 hpi). Scale bar, 1 μm. (**D**) Magnified view of the boxed region in (**C**). SMV, single membrane vesicle; DMV, double membrane vesicle. (**E**) RD cells stably expressing p97-GFP were mock-infected (upper panel) or infected with EV71 (MOI = 10) for 12 h (lower panel). The cells were then fixed and stained with rabbit anti-RTN3 and mouse anti-2C antibodies (p97, green; RTN3, red; 2C, purple; nuclei, blue).

To further observe the EV71-induced ROs in cells, electron microscopy was performed using the high-pressure freezing (HPF) and freeze-substitution (FS) methods [80–82]. In EV71-infected cells, large quantities of membranous vesicles accumulated in the perinuclear region of 100–300 nm in diameter, and were surrounded by single- or double-membranes. Some vesicles closely contacted the swelled ER (Fig 9A–9D), again indicating that the EV71-induced ROs may originate from the ER.

To confirm that the ER membranes and ER molecules were hijacked by EV71 to form viral ROs, the distribution of reticulon 3 (RTN3), an ER membrane molecule, was assessed using immunofluorescence [76,83,84]. The results showed that RTN3 was redistributed and concentrated in perinuclear foci and was colocalized with p97 and EV71 2C protein in EV71-infected cells (Fig 9E), suggesting that RTN3 was redistributed from the ER membrane to viral ROs during EV71 infection. Taken together, these data suggest that EV71-induced ROs are closely interrelated with the ER, and that the membranes of ROs originated from the ER. Thus, it is rational to infer that EV71 inhibits cellular ERAD, hijacks ERAD component p97, and takes advantage of ER-derived membrane components to benefit its own replication.

## Discussion

**Fig 10:**
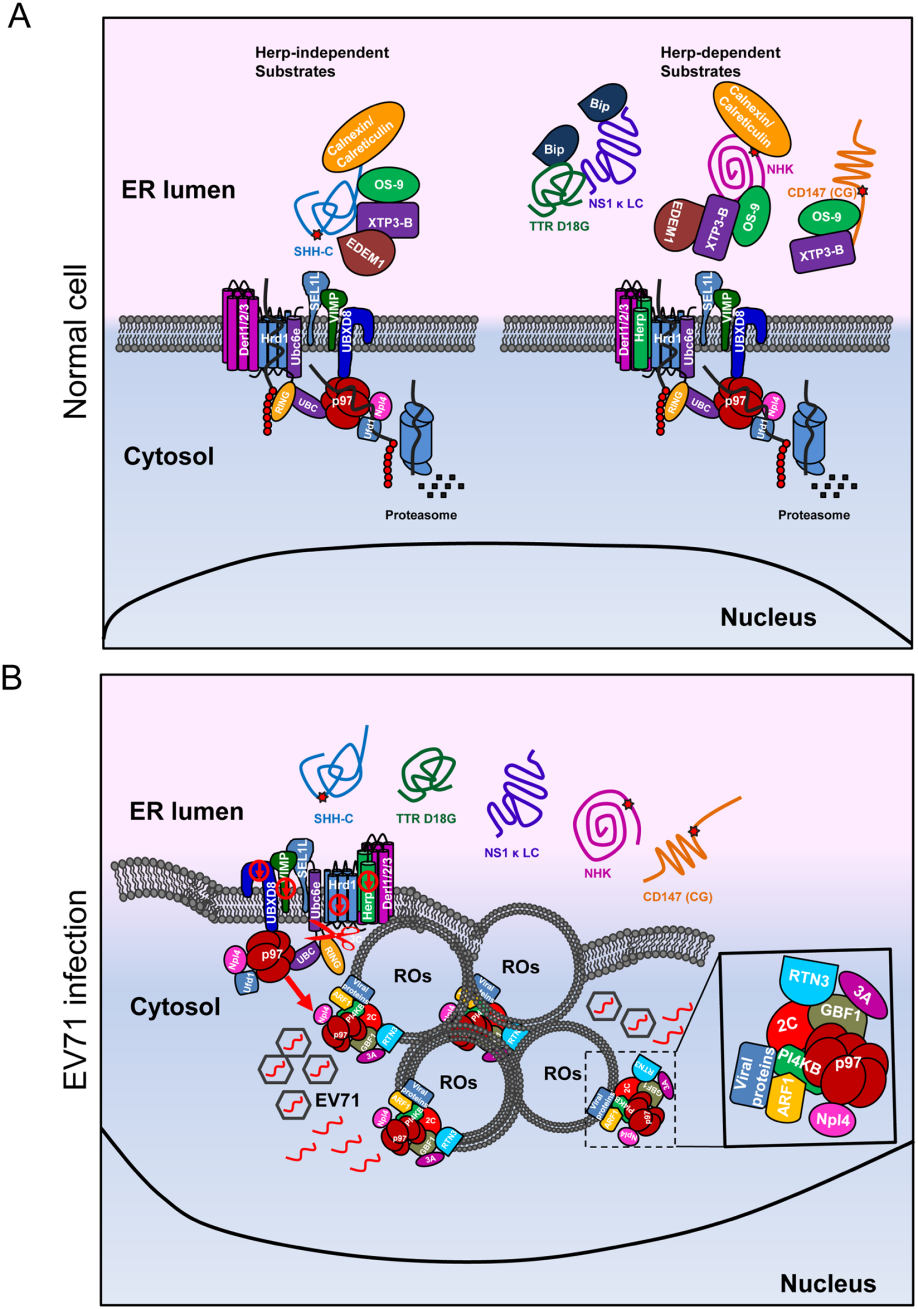
Model of the mechanism by which EV71 inhibits the ERAD pathway and hijacks p97 to promote its replication. (**A**) In normal cells, different substrates are degraded through different pathways: the calnexin/calreticulin substrate SHH-C is degraded through a Herp-independent pathway, and the calnexin/calreticulin substrate NHK and the BiP substrates TTR D18G, NS1κ LC, and endogenous substrate CD147 are degraded through a Herp-dependent pathway. (**B**) In EV71-infected cells, EV71 inhibits the ERAD pathway at multiple places: it downregulates the expression of Herp, Hrd1, VIMP, and UBXD8 and cleaves Ubc6e. The disabled ERAD causes different substrates to be tethered in the ER lumen. EV71 hijacks ER membranes to form single- or double-membrane vesicles. p97 is hijacked from disabled ERAD to form ROs and co-localizes with EV71 2C, RTN3, and other replication-related molecules including PI4KB, ARF1, and GBF1.

In this study, we extensively investigated the impact of EV71 on ERAD and reported a novel relationship between the virus and cellular ERAD. These findings are summarized in Fig 10.

In previous studies, all the reported relationships between viruses and cellular ERAD are common in that the viruses use the ERAD machinery for their own benefit [28,31–33]. However, EV71 uses a totally different strategy to influence cellular ERAD. It inhibits ERAD via viral proteases, thoroughly suppresses physiological ERAD at multiple points. EV71 then hijacks host factor p97 and utilizes ER-derived membrane components from the disabled ERAD machinery to form its own replication organelles. In this study, we found that EV71 viral protein expression and viral RNA replication did not change in Ubc6e or Herp knockdown cells, although degradation of ERAD substrates was inhibited. We thought this was because the damage to ER caused by Ubc6e or Herp knockdown was relatively mild when compared to EV71 infection, and the ER membranes were not rearranged under this situation. However, in EV71 infected cells, ERAD was inhibited at multiple points, and ER homeostasis suffered serious damage. This will inevitably cause changes to ER and lead to membrane rearrangement, allowing viruses to take advantage of ER membranes and ER-related host factors more conveniently [76,85].

This study revealed novel functions for the EV71 proteases 2A^pro^ and 3C^pro^. The picornavirus protease influences many cellular processes and previous studies have mainly focused on innate immunity and gene expression [5,13,86]. To our knowledge, this is the first study to reveal a role for viral proteases in the ERAD process. However, future studies are needed to determine whether other viral proteases have the same function.

During ERAD, the E2 ubiquitin-conjugating enzyme and E3 ligase work cooperatively to ubiquitinate substrates. To date, three E2s (Ubc6e/UBE2J1, UBE2J2, and UBE2G2) and five principal E3s (Hrd1, gp78, RMA1, TEB4, and TRC8) have been identified in the mammalian ERAD system [29,54–56,87]. Among these, Hrd1 forms an E3-E2 pair with Ubc6e and gp78 forms an E3-E2 pair with UBE2G2. These two E3-E2 pairs are considered the main executors of substrate ubiquitination in ERAD and they have distinct substrate specificities that are determined by the E3s [56]. The current results revealed that EV71 infection caused both Ubc6e cleavage and Hrd1 downregulation and thus totally destroyed the Hrd1-Ubc6e E3-E2 pair. We also investigated the gp78-UBE2G2 pair, but there were no obvious changes in these two molecules during EV71 infection (Fig 2B), indicating that the Ubc6e-Hrd1 E2-E3 pair is the key ubiquitination element targeted by EV71 during infection. We attempted to reconstitute a functional ERAD system in EV71-infected cells by co-transfecting cells with a 3C^pro^-resistant mutant of Ubc6e (Q219Q260Q273A) and WT Hrd1. However, this could not rescue the inhibited ERAD process (S8 Fig), suggesting that EV71 inhibits ERAD in a comprehensive manner that involves multiple factors. The decrease of Hrd1 and the cleavage of UBXD8 found in our study are examples. We also checked the role of EV71 2A^pro^ and 3C^pro^ in the cleavage of UBXD8 but the results excluded their role (S4 Fig) and our future work will focus on the specific mechanisms.

In this study, we identified Ubc6e as the key E2 ubiquitin-conjugating enzyme in EV71-disturbed ERAD. Knockdown of Ubc6e by siRNA inhibited the degradation of all ERAD substrates we tested. However, in one recent study, Hagiwara et al. reported accelerated degradation of NHK in Ubc6e−/− cells because Ubc6e could downregulate ERAD enhancers [88]. This discrepancy might be caused by the different silencing efficiencies of the methods used by the two groups. We used siRNA to knock down Ubc6e while Hagiwara et al. used Ubc6e knock-out MEFs. As described by Hagiwara et al. themselves, very little UBC6e suffices to properly regulate ERAD enhancers [88]. When using siRNA, knockdown of Ubc6e was not complete, and the remaining Ubc6e was sufficient to maintain the proper expression level of ERAD enhancers. Moreover, in Ubc6e−/− cells, other E2s may compensate in the Hrd1 complex and substitute Ubc6e to degrade ERAD substrates.

Okuda-Shimizu et al. and Kny et al. reported conflicting results about the role of Herp in the degradation of different substrates [42,50]. The current study explored the involvement of Herp in different ERAD pathway and the results are consistent with Kny and colleagues. Specifically, Herp was involved in the ERAD of the BiP substrates NS1 κ LC and TTRD18G and the calnexin substrate NHK, but not another calnexin substrate SHH-C. This suggests that other signatures but not the binding chaperone and glycosylation determine the substrate specificity of Herp. As a critical constituent of ERAD, its turnover is mediated by ERAD tuning, and this is a strategy by which Herp can regulate ERAD [64,89]. A previous study demonstrated that UBE2G2-gp78 is responsible for Herp degradation during the recovery from ER stress [63]. However, the mechanism by which Herp is degraded under physiological conditions is unknown. In the current study, Ubc6e was identified as the key E2 that participates in Herp degradation since silencing Ubc6e increased the intracellular levels of Herp (Fig 6A–6E) and silencing UBE2G2 had no effect (S9A Fig). We also tried to identify the E3 partner of Ubc6e that participates in Herp degradation. Although the candidate E3s included Hrd1, gp78, and RNF5, we unfortunately could not draw a definite conclusion because Herp was not upregulated when any of them were silenced (S9A–S9C Fig). Nevertheless, we speculate that Hrd1 is involved in the degradation of Herp because overexpressing a dominant-negative mutant of Hrd1 (C329S) increased Herp expression compared with WT Hrd1 (S9D Fig). However, it is unclear why silencing Hrd1 had little impact on Herp whereas overexpressing Hrd1 dramatically increased Herp levels. We postulate that Hrd1 and its association with Herp is a key determinant of intracellular Herp expression. Specifically, when Hrd1 is silenced Herp is downregulated, but when Hrd1 is overexpressed Herp is upregulated. Future studies are needed to verify this hypothesis and clarify the specific mechanism by which Herp is degraded.

Previous studies demonstrated that p97 is a host factor for viral replication. Arita et al. demonstrated that p97 is required for poliovirus replication and is involved in the cellular protein secretion pathway [73]. Panda et al. identified p97 as a conserved regulator of Sindbis virus entry [90]. A recent study demonstrated that p97 is essential to HCV replication and proposed that p97 is involved in the assembly of HCV replicase [91]. In addition, Wu et al. identified p97 as a cellular factor involved in EV71 replication using a genome-wide RNAi screen [92]. The current study proposed that EV71 hijacks p97 from the disabled ERAD machinery, identified p97 as a host factor for EV71 replication, and demonstrated that p97 co-localized with the viral protein 2C on EV71-induced ROs. This study supports the role of p97 in the viral lifecycle and discloses the origin of the p97 that participates in the formation of ROs. However, it remains unclear how p97 functions in ROs. We propose that p97 might participate in EV71 replication complex assembly and membrane rearrangement via its AAA+ATPase activity.

In this study, we tried to distinguish EV71-induced ROs from EDEMosomes. According to previous reports, virus-induced ROs originating from EDEMosomes have some common features: they are double-membraned vesicles coated with EDEM1 and nonlipidated LC3; silencing LC3 significantly inhibits virus propagation [32,35,37,93,94]. However, EV71-induced ROs we observed under electron microscopy included both single- and double-membrane vesicles, and most were single-membrane vesicles. We also checked the distribution of EDEM1 in EV71-infected cells, and the results showed that it did not change during EV71 infection (S10 Fig). Therefore, we conclude that EV71-induced ROs differ from EDEMosomes and that EV71 influences the cellular ERAD machinery via a totally novel and different mechanism.

Previous studies revealed that picornavirus formed replication organelles at ER-Golgi interface and took advantages of membranes and host factors of both ER and Golgi origin [74–79,82,83,95–99], but it was unclear why picornavirus tend to choose these specific elements of the host cells for their replication and how they are utilized. The current study found that EV71 inhibited cellular ERAD, a critical function through which the ER maintains homeostasis. Inhibiting ERAD inevitably causes ER deformation, which may lead to membrane rearrangement and allows viruses to take advantage of host membranes and host factors more conveniently.

Taken together, this study demonstrated a novel relationship between EV71 and the cellular ERAD system. p97 was identified as a new host factor that is essential for EV71 replication; it redistributed and co-localizes with the EV71 viral proteins in EV71-induced ROs. These findings provide potential targets for the anti-viral treatment of EV71 infections.

## Materials and methods

### Cell lines, viruses, and stable cell line generation

Rhabdomyosarcoma (RD) cells and human embryonic kidney 293T (HEK-293T) cells were purchased from ATCC. They were cultured in MEM (Modified Eagle’s medium) and DMEM (Dulbecco’s modified Eagle’s medium), respectively, supplemented with 10% fetal bovine serum (FBS) and penicillin (100 units/ml)/streptomycin (100 mg/ml). BSRT7 cells were described in our previous study [4] and cultured in DMEM supplemented with 10% FBS and 1 mg/ml G418. All cells were maintained at 37°C in a humidified atmosphere of 5% CO_2_ and 95% air. EV71 is a Fuyang strain (GenBank accession no. FJ439769.1) and was propagated in RD cells. RD cells stably expressing SHH, NHK, TTR D18G, and NS1 κ LC were obtained by transfecting RD cells with the corresponding plasmids followed by selection using 1 mg/ml G418.

### Antibodies and reagents

The following antibodies were used in this study: anti-β-actin (A5441), anti-GFP (GSN24), anti-HA (H6908), anti-FLAG (A8592), anti-V5 (V8012), anti-RNF5 (SAB2701502), anti-EDEM1 (E8406), anti-OS9 (SAB4200021), anti-Derl1 (D4443), anti-Derl2 (D1194) and anti-calnexin (C4731) were purchased from Sigma-Aldrich; anti-Hrd1 (12925S), anti-Npl4 (13489S), anti-BiP (3183S), anti-calreticulin (2891S), anti-VIMP (15160S), anti-eIF4GI (2858S), and anti-gp78 (9590S) were purchased from Cell Signaling Technology; anti-EV71 3C (GTX630191) was purchased from Genetex; anti-CD147 (11989-1-AP), anti-c-MYC (10828-1-AP), anti-PI4KB (13247-1-AP), anti-ARF1 (20226-1-AP), anti-GBF1 (25183-1-AP), and anti-RTN3 (12055-2-AP) were purchased from Proteintech; anti-UBE2G2 (ab174296), anti-XTP3-B (ab181166), anti-UBXD8 (ab154064), anti-Ufd1 (ab181080), and anti-p97 (ab11433) were purchased from Abcam; anti-SEL1L (sc-48081), anti-Herp (BML-PW9705), and anti-Ubc6e (TA504988) were obtained from Santa Cruz Biotechnology, Enzo Life Sciences, and Origene, respectively; anti-EV71 (MAB979) and anti-EV71 VP1 (MAB1255-M05) were purchased from Millipore and Abnova, respectively. The corresponding IRDye 680- or 800-labeled secondary antibodies were obtained from LI-COR Biosciences. The fluorescence-labeled secondary antibodies used in immunostaining were purchased from Jackson ImmunoResearch. EV71 2C antibodies were generated in rabbits or mice using recombinant protein as the immunogen. DBeQ (SML0031), CHX (C4859), DTT (D9779), and Tg (T9033) were purchased from Sigma-Aldrich. DAPI (D1306), Lipofectamine 2000, and Lipofectamine RNAiMAX were purchased from Invitrogen. Tun (12819) and MG132 (474790) were purchased from Cell Signaling Technology and Calbiochem, respectively. G418 (E859) was purchased from Amresco. Lambda protein phosphatase (P0753) was purchased from New England Biolabs.

### Plasmids

pCMV6-SHH, pCMV6-TTR and pCMV6-Ubc6e were purchased from Origene. NHK cDNA were kindly provided by Dr. Richard N. Sifers (Baylor College of Medicine, USA). NS1 κ LC plasmid was a gift from Dr. Linda M. Hendershot (St. Jude Children’s Research Hospital, USA). The pCMV-SP-S11-NHK-HA and pRRL-S1–10 constructs were gifts from Dr. Shengyun Fang (University of Maryland School of Medicine, USA). pcDNA3.1-IRES-2A was a generous gift from Dr. Shih-Yen Lo (Tzu Chi University, Taiwan, China). To construct pCMV6-NHK and pCMV6-NS1 κ LC, cDNA templates were amplified by PCR and then cloned into the *Sgf*I and *Mlu*I sites of pCMV6-Entry vector. To construct pCMV-SHH-S11-HA and pCMV-TTR D18G-MycFlag-S11, SHH, TTR D18G, and linker-S11 sequences were amplified by PCR and then cloned into the *Eco*RI and *Sal*I sites of pCMV-SP-S11-NHK-HA. To construct the pVRC-p97-FLAG plasmid, RNA from RD cells was reverse transcribed into cDNA, and p97 cDNA was amplified by PCR and cloned into the *Sal*I and *Xba*I sites of pVRC. To construct pCMV6-p97-GFP, the p97 sequence was amplified from pVRC-p97-FLAG by PCR and then cloned into the *Sgf*I and *Mlu*I sites of pCMV6-AC-GFP. To construct pVRC-2C-HA and pVRC-3C-FLAG, the 2C and 3C cDNAs were amplified by PCR from pEGFPC1-2C/3C and then cloned into the *Sal*I and *Xba*I sites of pVRC vector. pCMV6-TTR D18G, pVRC-p97QQ-FLAG, and plasmids expressing Ubc6e mutants were generated by site-directed mutagenesis. pcDNA3.1-IRES-2A(C110A) and pEGFPC1-3C/3C(C147S) were described previously [4,100].

### Immunoblotting

Cells were lysed on ice for 30 min in lysis buffer (25 mM Tris-Cl, 150 mM NaCl, 1 mM EDTA, 1% NP-40 or 1% Triton X-100, pH 7.4) supplemented with protease inhibitor cocktail (Roche, 04693132001). Then, the cell lysates were centrifuged at 20,000× *g* at 4°C for 15 min to remove insoluble materials. Equal amounts of total proteins (20–100 μg) were separated by 8%–15% SDS-PAGE and then transferred to nitrocellulose membranes (Pall, 66485). After blocking with 5% nonfat dry milk solution in TBS at room temperature for 1 h, the membranes were incubated with different primary antibodies overnight at 4°C. The following day the membranes were washed three times in TBST and then incubated with IRDye 680 or 800-labeled secondary antibodies at room temperature for 2 h. They were then scanned using the Odyssey Infrared Imaging System (LI-COR Biosciences), and immunoblot bands were quantified using Image J software (National Institutes of Health).

#### Co-immunoprecipitation

Cells were lysed on ice for 30 min in lysis buffer (25 mM Tris-Cl, 150 mM NaCl, 1 mM EDTA, 1% NP-40 or 1% Triton X-100, pH 7.4). Equal amounts of cell lysates were incubated with the appropriate antibodies at 4°C overnight. The following day, protein A-agarose beads (Sigma-Aldrich, P2545) were washed and added to the cell lysates and incubated for an additional 4 h at 4°C. Then, immunoprecipitates were washed once with lysis buffer and three times with TBS. Finally, the agarose beads were boiled in 2× SDS sample buffer and analyzed by western blotting with the corresponding antibodies.

### Cycloheximide chase assays

RD cells or RD cells stably expressing SHH, NHK, TTR D18G, and NS1 κ LC were incubated with 100 μg/ml CHX in culture medium at 37°C for the indicated times to inhibit *de novo* protein synthesis. Then, the cells were collected and lysed in ice-cold lysis buffer for further analysis.

### *In vitro* cleavage assay

The recombinant EV71 3C^pro^ and 3C protease-dead mutant 3C^pro^(E71A) were previously described [101,102] and provided by Dr. Sheng Cui (Institute of Pathogen Biology, Chinese Academy of Medical Sciences & Peking Union Medical College, China). In the *in vitro* cleavage assay, 293T cell lysates were incubated with indicated amounts of 3C^pro^ or 3C^pro^(E71A) in reaction buffer (50 mM Tris-HCl, pH 7.0, 200 mM NaCl) at 30°C for 2 h. Then, the mixtures were analyzed by immunoblotting.

### Transfection of plasmids and siRNAs

Cells were transfected with plasmids or siRNA duplexes using Lipofectamine 2000 (Invitrogen, 11668019) and Lipofectamine RNAiMAX (Invitrogen, 13778100), respectively, according to the manufacturer’s instructions. siRNAs were transfected at a final concentration of 40 nM. siRNAs were purchased from Guangzhou RiboBio and the sequences are shown in S1 Table. Herp siRNA (sc-75245) was purchased from Santa Cruz Biotechnology with unknown sequences.

### Immunofluorescence and confocal microscopy

RD cells were fixed with 4% paraformaldehyde in PBS for 15 min and permeabilized using 0.3% Triton X-100 in PBS containing 3% BSA (Sigma-Aldrich, A7906) for 30 min. After washing three times with PBS, the cells were incubated with primary antibodies overnight at 4°C. The following day, cells were incubated with the appropriate fluorescence-conjugated secondary antibodies (Jackson ImmunoResearch) for 1 h at room temperature followed by DAPI for nuclear staining. Images were captured and analyzed using a TCS SP5 laser-scanning confocal microscope and LAS AF software (Leica Microsystems).

### Real-time PCR

Total cellular RNA was extracted using Trizol reagent (Life Technologies, 15596–018) according to the manufacturer’s protocols. One microgram of total RNA was reverse transcribed in a 20 μl volume using Improm-IITM reverse transcriptase (Promega, A3500) following the manufacturer’s instructions. Real-time PCR was performed using 1 μl cDNA as the template with PowerUp SYBR Green Master Mix (Applied Biosystems, Life Technologies, A25742). The reaction was run on a 7900HT Fast Real-Time PCR machine (Applied Biosystems, Life Technologies), and levels of gene mRNAs were normalized to GAPDH mRNA. Results were analyzed as fold-change using the ΔΔC_T_ method. The primers for EV71 RNA quantification were described previously [100], and the sequences of other primers were as follows:

Herp F for RD cells: 5´-CCGGTTACACACCCTATGGG-3´

Herp R for RD cells: 5´-TGAGGAGCAGCATTCTGATTG-3´

GAPDH F for RD cells: 5´-AAAATCAAGTGGGGCGATGCT-3´

GAPDH R for RD cells: 5´-GGGCAGAGATGATGACCCTTT-3´

VIMP F for BSRT7 cells: 5´-TGAGATTCCTGCACGTCACAGT-3´

VIMP R for BSRT7 cells: 5´-AGTGCCCTCAGTCGGACTGTA-3´

GAPDH F for BSRT7 cells: 5´-GACCTCAACTACATGGTCTAC-3´

GAPDH R for BSRT7 cells: 5´-TCGCTCCTGGAAGATGGTGAT-3´

### High-pressure freezing and transmission electron microscopy (TEM)

RD cells grown on 10-cm dishes were mock-infected or infected with EV71 (MOI = 10) for 12 h. The cells were harvested and washed three times with PBS and then frozen using a high-pressure freezer (Leica EM HPM100). Freeze substitutions were performed in a substitution unit (Leica EM AFS2) in dry acetone with 2% osmium tetroxide at −90°C for 72 h followed by gradual warming to −20°C for 8 h and 0°C for 2 h. After washing with dry acetone at 0°C three times, the samples were warmed to room temperature. After infiltration with SPI812 resin for several hours, the samples were embedded into SPI812 resin, placed in a 60°C oven for 24 h, and then sectioned using a microtome (Leica EM UC6). Ultrathin sections were stained with uranyl acetate and lead citrate and then examined by transmission electron microscopy (FEI Tecnai Spirit 120 KV).

## Supporting information

S1 Fig EV71 infection inhibits the degradation of CNX/CRT-dependent glycosylated ERAD substrates. (**A, B**) RD cells stably expressing SHH-FLAG (**A**) or NHK-FLAG (**B**) were mock-infected (−) or infected (+) with EV71 (MOI = 10) for 6 h and then treated with 10 μg/ml tunicamycin (Tun) for an additional 2, 4, or 6 h. The cells were then harvested and cell lysates were analyzed by western blotting with the indicated antibodies. (**C**) RD cells were mock-infected or infected with EV71 (MOI = 10) for 9 h, and the cells were then treated with or without CHX (100 μg/ml) for another 8 h. The apoptosis of cells was then analyzed by flow cytometry. The Annexin V-positive and PI-negative cells were considered to be apoptotic in the early phase, and the annexin V-positive and PI-positive cells were considered to be apoptotic in the late phase.
(TIF)

S2 Fig TTR D18G contains glycosylated and deglycosylated forms. RD cells stably expressing TTR D18G-FLAG were treated with CHX (100 μg/ml) for the indicated times, and cell lysates were digested with PNGase F at 37°C for 30 min. The lysates were then analyzed by western blotting with FLAG antibodies; actin was used as the loading control. Arrows indicate glycosylated and non-glycosylated TTR D18G, respectively.
(TIF)

S3 Fig Apoptosis in cells infected with EV71 for 18 h. RD cells were mock-infected or infected with EV71 (MOI = 10) for 10 h, then the cells were treated with or without MG132 (50 μM) for another 8 h. Apoptosis was analyzed by flow cytometry. Annexin V-positive and PI-negative cells were considered to be apoptotic in the early phase, and annexin V-positive and PI-positive cells were considered to be apoptotic in the late phase.
(TIF)

S4 Fig EV71 2A^pro^ and 3C^pro^ were not involved in the cleavage of UBXD8. (**A**) BSRT7 cells were transfected with empty vector or increasing doses of pcDNA3.1-IRES-2A (1–4 μg). At 36 h post-transfection, cells were harvested and cell lysates were analyzed by western blotting with antibodies against UBXD8, eIF4GI, and V5. (**B**) 293T cells were transfected with plasmids encoding GFP or GFP-3C. At 36 h post-transfection, cells lysates were analyzed by western blotting with antibodies against UBXD8 and GFP.
(TIF)

S5 Fig The viral protease 2A^pro^ cannot cleave Ubc6e. 293T cells were first transfected with a plasmid encoding T7 RNA polymerase. At 24 h after transfection, cells were re-transfected with increasing doses (0–4 μg) of pcDNA3.1-EGFP or pcDNA3.1-IRES-2A plasmid. At 36 h after transfection, cell lysates were analyzed by western blotting with antibodies against Ubc6e (mouse monoclonal) and 2A-V5; actin was used as an internal control.
(TIF)

S6 Fig Apoptosis in cells infected with EV71 combined with treatment of other chemicals. RD cells were mock-infected or infected with EV71 (MOI = 10) for 9 h and then treated with MG132 (50 μM), Tg (300 nM), Tg plus MG132, Tun (10 μg/ml), or Tun plus MG132 for an additional 6 h. Apoptosis was then analyzed by flow cytometry. Annexin V-positive and PI-negative cells were considered to be apoptotic in the early phase, and annexin V-positive and PI-positive cells were considered to be apoptotic in the late phase.
(TIF)

S7 Fig VIMP has a very short half-life. RD cells were treated with CHX (100 μg/ml) for 4 h. Cell lysates were then separated by SDS-PAGE and western blotting was performed using VIMP and Herp antibodies. Herp expression served as a control molecule with a short half-life.
(TIF)

S8 Fig Overexpression of the Ubc6e 3C^pro^-resistant mutant together with Hrd1 cannot rescue the degradation of SHH-C during EV71 infection. RD cells stably expressing SHH-FLAG were transfected with empty vector (control) or the Ubc6e triple-site mutant pVRC-Ubc6e-Q219Q260Q273A together with wild-type Hrd1. At 36 h post-transfection, mock infected (−) or infected (+) with EV71 (MOI = 10) for 12 h and then treated with (+) or without (−) CHX for 4 h. The cells were then harvested and the resulting cell lysates were analyzed by western blotting with the indicated antibodies.
(TIF)

S9 Fig Hrd1 is involved in Herp degradation. (**A**, **B**) RD cells were transfected with control and siRNA targeting UBE2G2 and gp78 (**A**), and RNF5 (**B**). At 36 h post-transfection, cells were mock-infected (−) or infected (+) with EV71 (MOI = 10) for 12 h and then treated with (+) or without (−) CHX for an additional 4 h. The cells were then harvested and cell lysates were analyzed by western blotting with the indicated antibodies. (**C**) RD cells were transfected with control or Hrd1 siRNA. At 36 h post-transfection, the cells were mock infected (−) or infected (+) with EV71 (MOI = 10) for 9 and 12 h, respectively. CHX-chased cells (100 μg/ml, 4 h) were used as a control for Herp downregulation. The cells were harvested and cell lysates were analyzed by western blotting with the indicated antibodies. (**D**) RD cells were transfected with empty vector, wide-type Hrd1, and Hrd1 C329S (dominant negative mutant). Thirty-six hours after transfection, cells were mock infected (−) or infected (+) with EV71 (MOI = 10) for 12 h. The cells were harvested and cell lysates were analyzed by western blotting with the indicated antibodies.
(TIF)

S10 Fig EDEM1 distribution is not changed in EV71 infected cells. RD cells were mock-infected or infected with EV71 (MOI = 10) for 12 h. Then, immunostaining was performed to detect the intracellular distribution of EDEM1 and EV71 2C (EDEM1, red; 2C, green; nuclei, blue). The insets show magnified views of merged channels in the boxed region.
(TIF)

S1 Table Sequences of siRNA duplexes used in this study. (DOCX)

S1 Text Supporting materials and methods. (DOCX)
