# Supplementary figures and images for: Enterovirus 71 protease 2Apro and 3Cpro differentially inhibit the cellular endoplasmic reticulum-associated degradation (ERAD) pathway via distinct mechanisms, and enterovirus 71 hijacks ERAD component p97 to promote its replication
Source: PLoS Pathog. 2017 Oct 6;13(10):e1006674. doi: 10.1371/journal.ppat.1006674 (PMC5650186; doi:10.1371/journal.ppat.1006674)

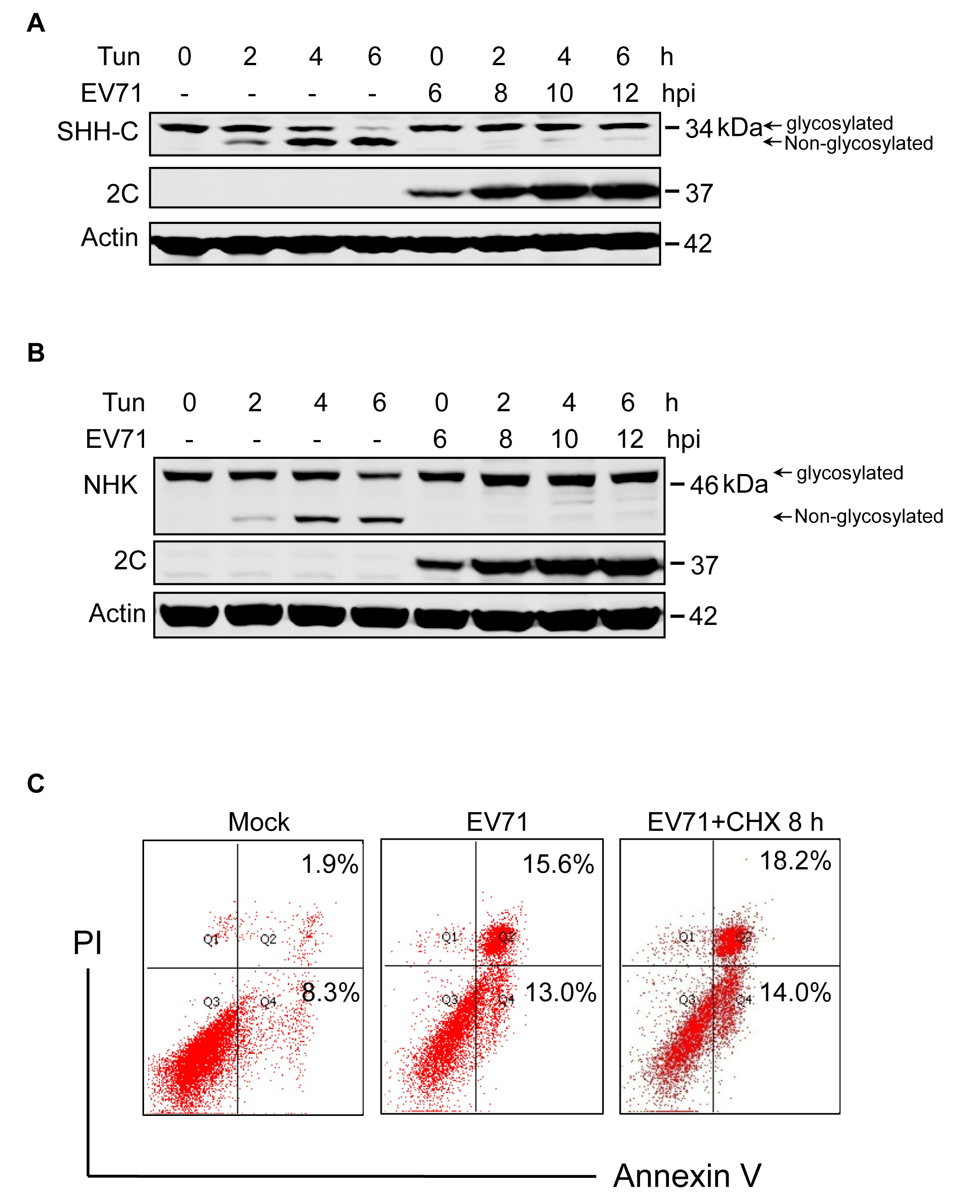

Supplement: S1 Fig — (A, B) RD cells stably expressing SHH-FLAG (A) or NHK-FLAG (B) were mock-infected (−) or infected (+) with EV71 (MOI = 10) for 6 h and then treated with 10 μg/ml tunicamycin (Tun) for an additional 2, 4, or 6 h. The cells were then harvested and cell lysates were analyzed by western blotting with the indicated antibodies. (C) RD cells were mock-infected or infected with EV71 (MOI = 10) for 9 h, and the cells were then treated with or without CHX (100 μg/ml) for another 8 h. The apoptosis of cells was then analyzed by flow cytometry. The Annexin V-positive and PI-negative cells were considered to be apoptotic in the early phase, and the annexin V-positive and PI-positive cells were considered to be apoptotic in the late phase. (TIF) [file ppat.1006674.s001.tif]

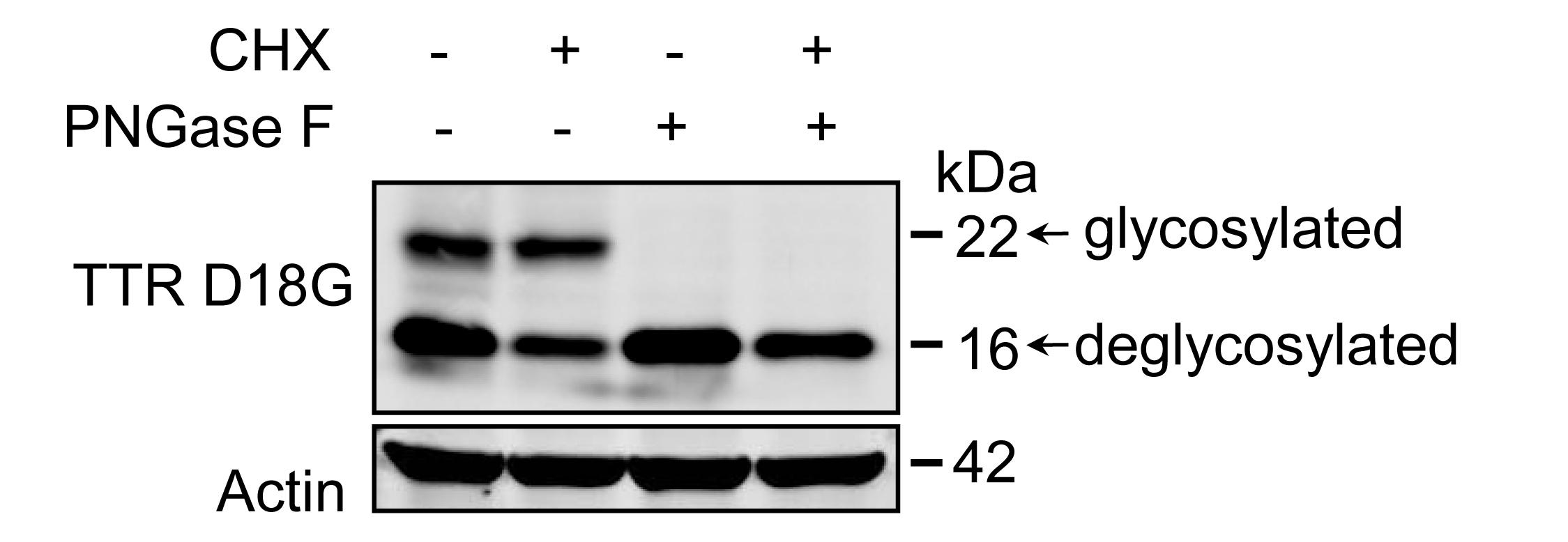

Supplement: S2 Fig — RD cells stably expressing TTR D18G-FLAG were treated with CHX (100 μg/ml) for the indicated times, and cell lysates were digested with PNGase F at 37°C for 30 min. The lysates were then analyzed by western blotting with FLAG antibodies; actin was used as the loading control. Arrows indicate glycosylated and non-glycosylated TTR D18G, respectively. (TIF) [file ppat.1006674.s002.tif]

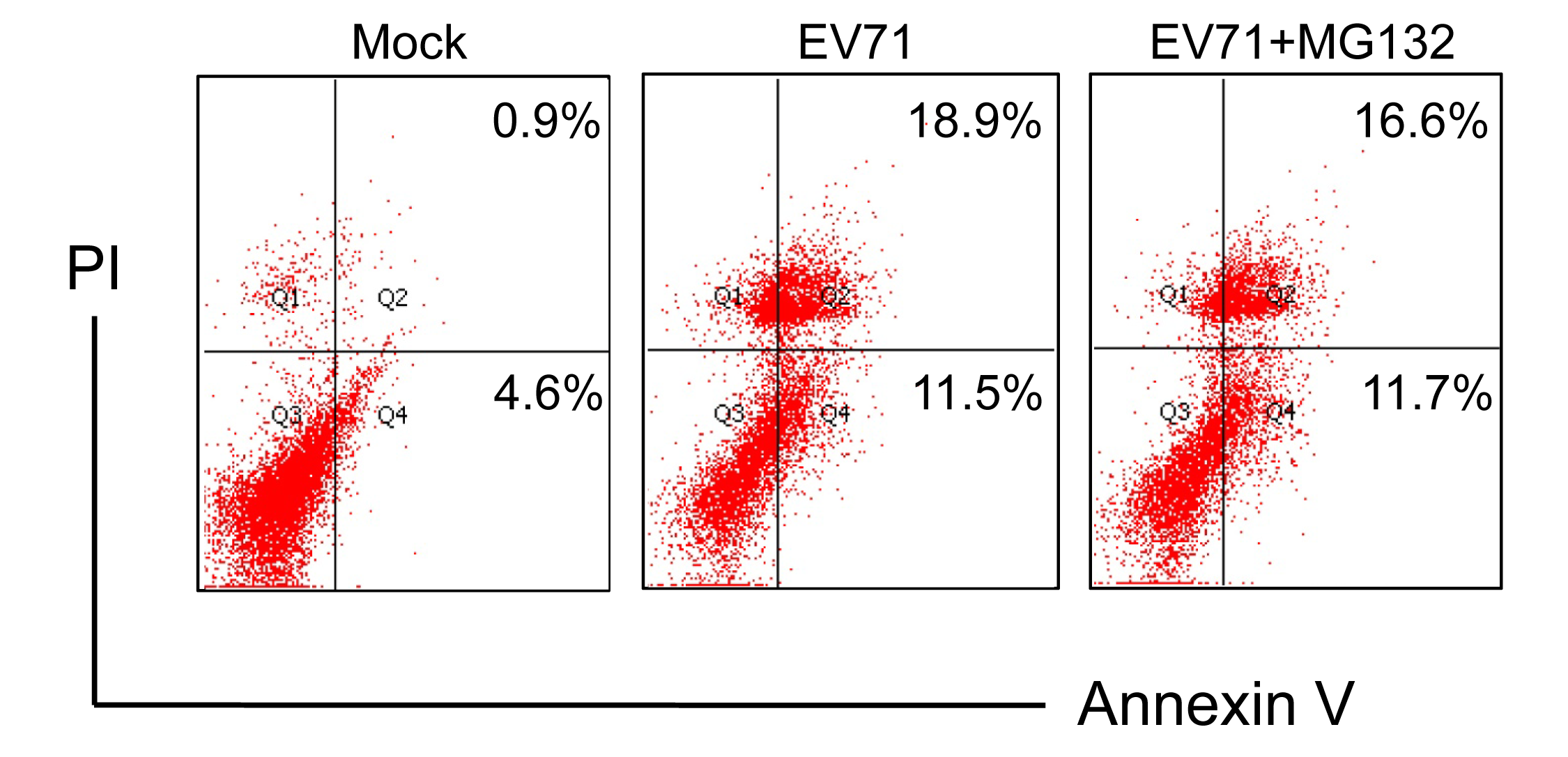

Supplement: S3 Fig — RD cells were mock-infected or infected with EV71 (MOI = 10) for 10 h, then the cells were treated with or without MG132 (50 μM) for another 8 h. Apoptosis was analyzed by flow cytometry. Annexin V-positive and PI-negative cells were considered to be apoptotic in the early phase, and annexin V-positive and PI-positive cells were considered to be apoptotic in the late phase. (TIF) [file ppat.1006674.s003.tif]

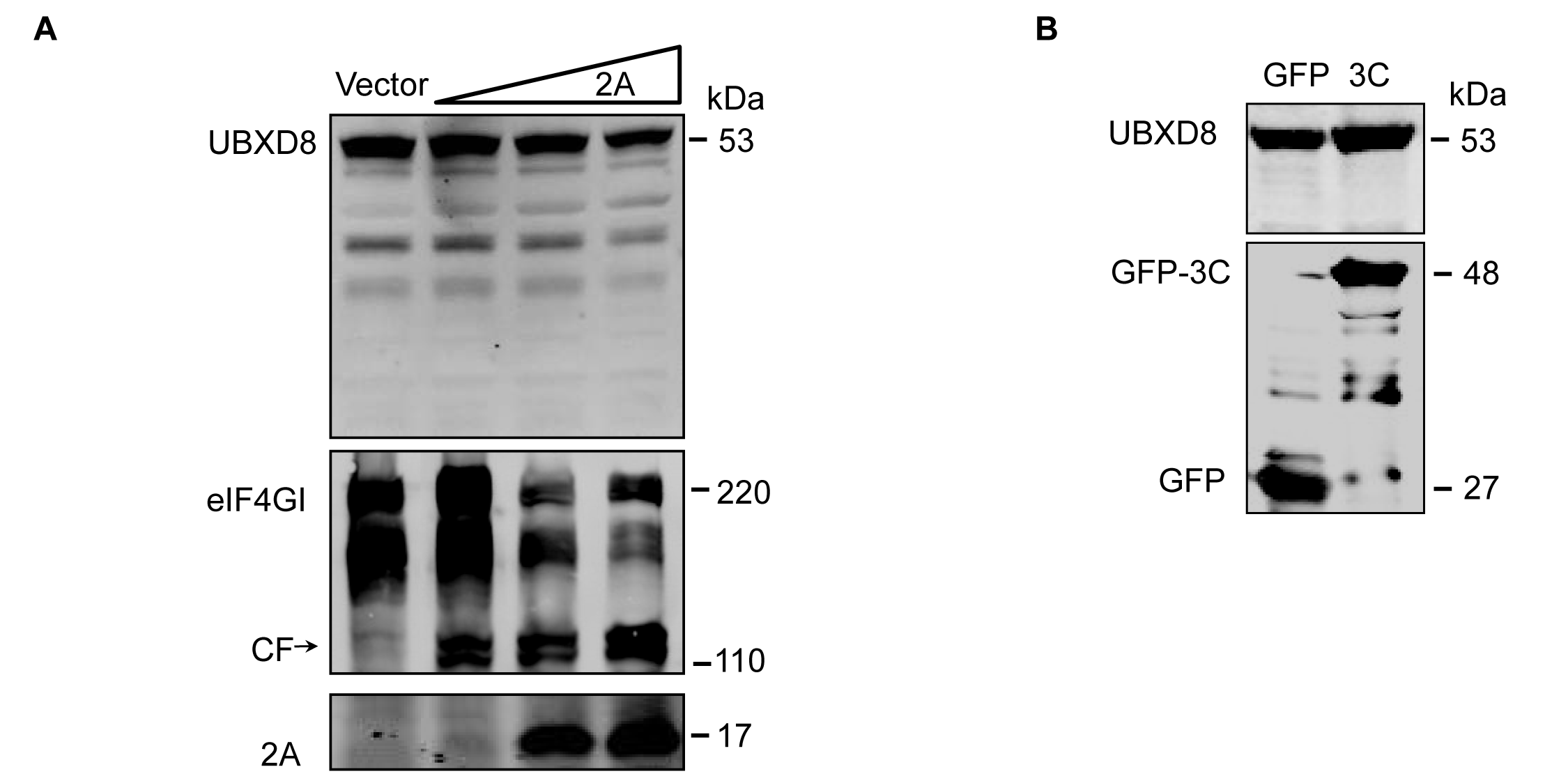

Supplement: S4 Fig — (A) BSRT7 cells were transfected with empty vector or increasing doses of pcDNA3.1-IRES-2A (1–4 μg). At 36 h post-transfection, cells were harvested and cell lysates were analyzed by western blotting with antibodies against UBXD8, eIF4GI, and V5. (B) 293T cells were transfected with plasmids encoding GFP or GFP-3C. At 36 h post-transfection, cells lysates were analyzed by western blotting with antibodies against UBXD8 and GFP. (TIF) [file ppat.1006674.s004.tif]

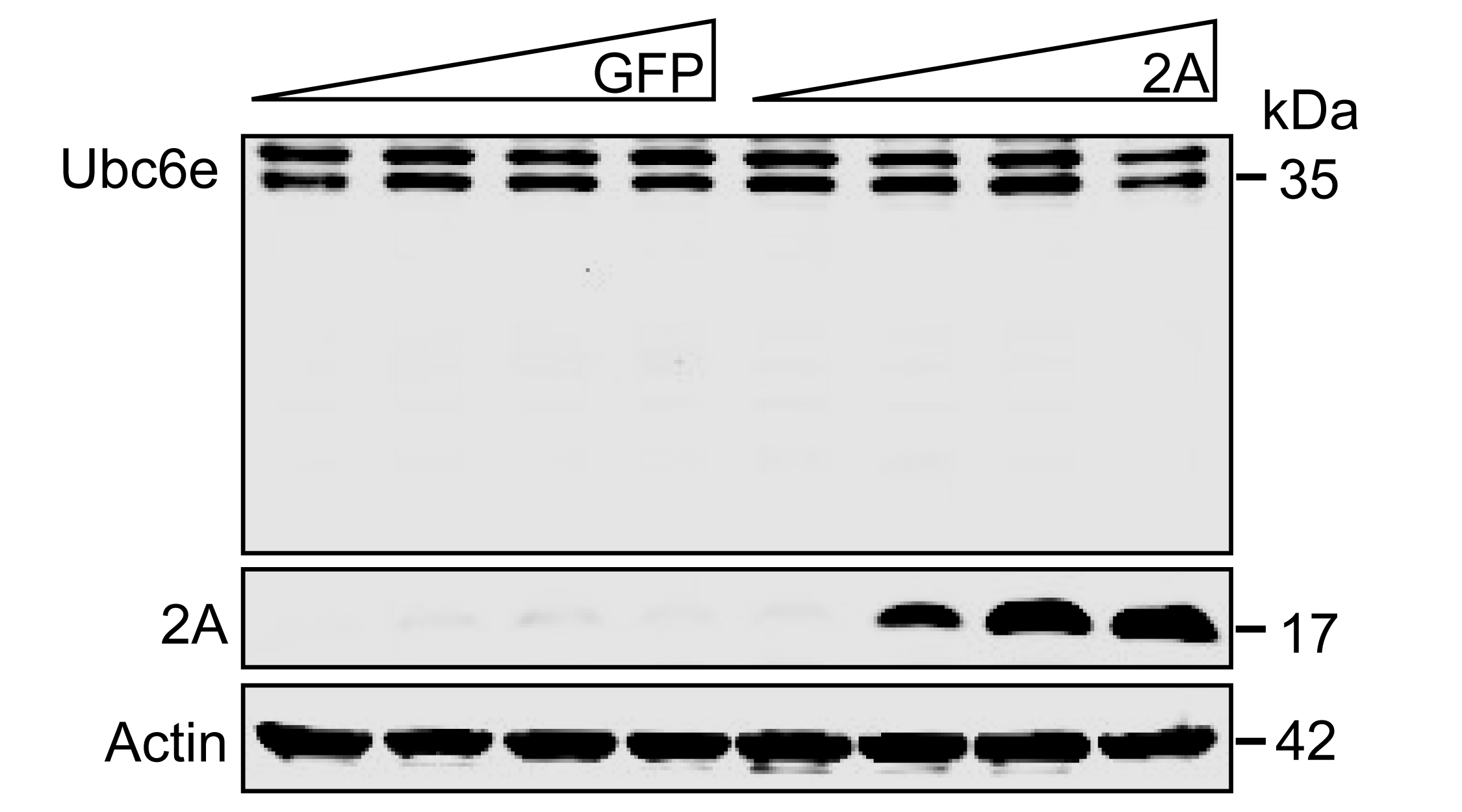

Supplement: S5 Fig — 293T cells were first transfected with a plasmid encoding T7 RNA polymerase. At 24 h after transfection, cells were re-transfected with increasing doses (0–4 μg) of pcDNA3.1-EGFP or pcDNA3.1-IRES-2A plasmid. At 36 h after transfection, cell lysates were analyzed by western blotting with antibodies against Ubc6e (mouse monoclonal) and 2A-V5; actin was used as an internal control. (TIF) [file ppat.1006674.s005.tif]

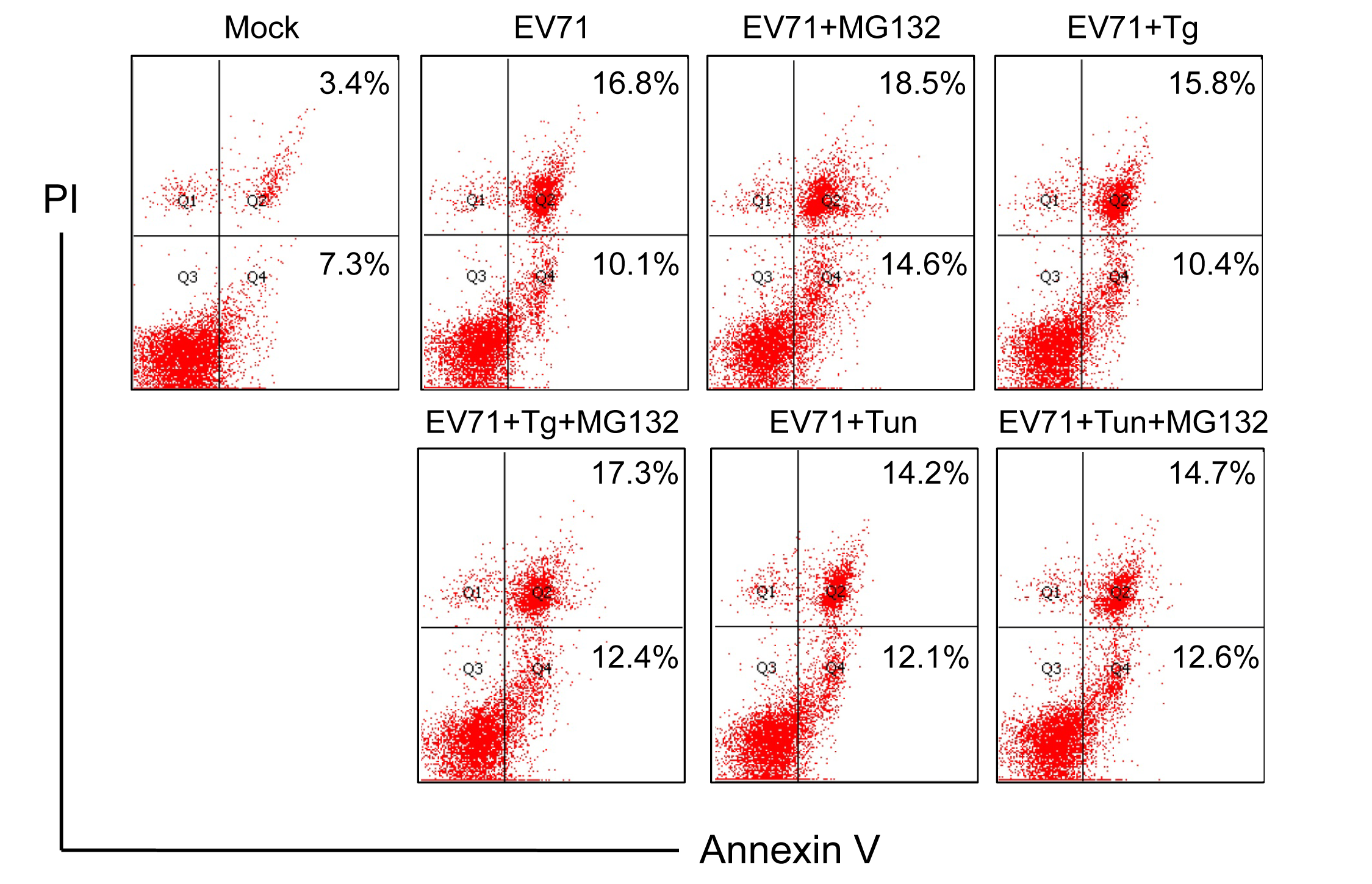

Supplement: S6 Fig — RD cells were mock-infected or infected with EV71 (MOI = 10) for 9 h and then treated with MG132 (50 μM), Tg (300 nM), Tg plus MG132, Tun (10 μg/ml), or Tun plus MG132 for an additional 6 h. Apoptosis was then analyzed by flow cytometry. Annexin V-positive and PI-negative cells were considered to be apoptotic in the early phase, and annexin V-positive and PI-positive cells were considered to be apoptotic in the late phase. (TIF) [file ppat.1006674.s006.tif]

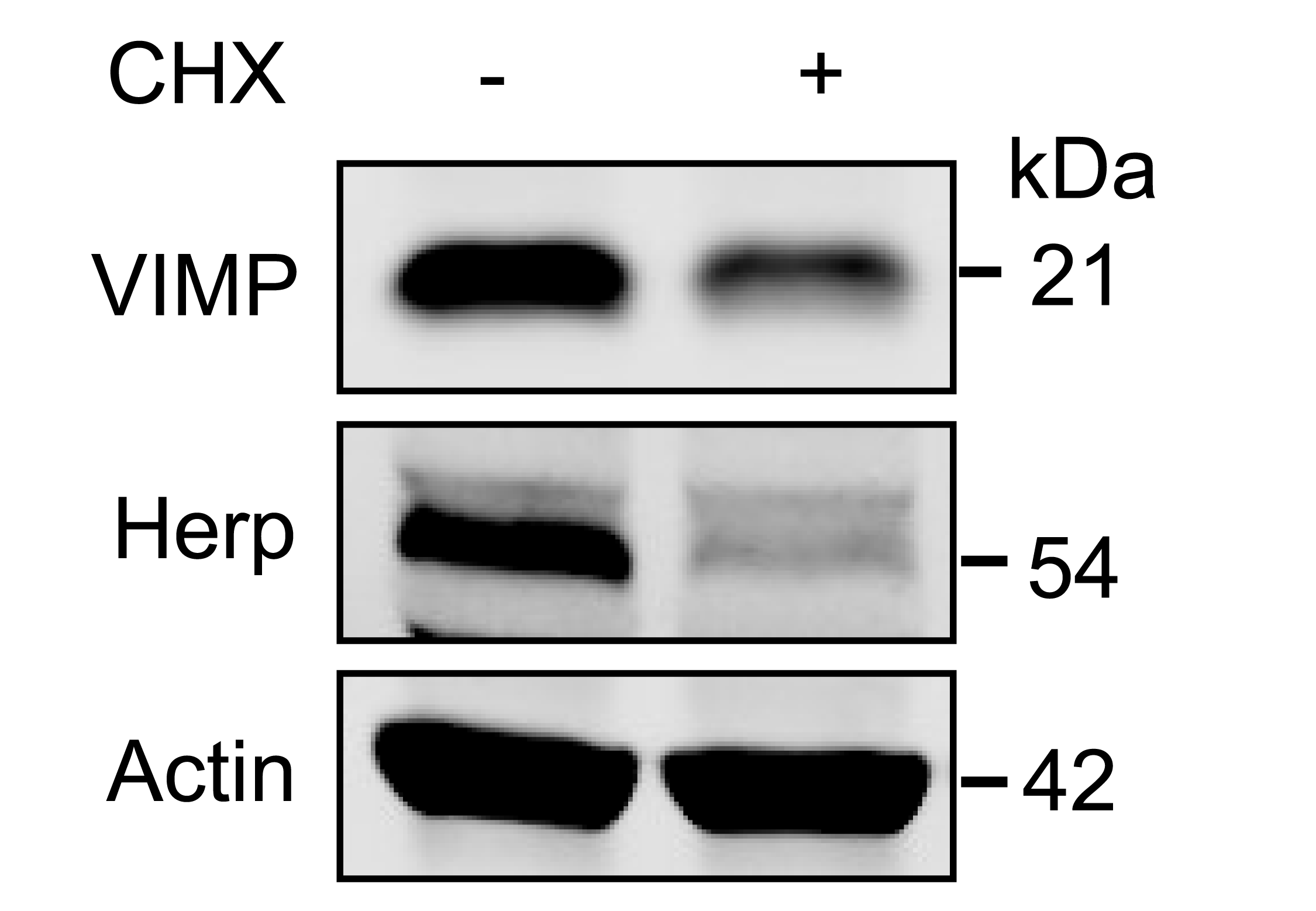

Supplement: S7 Fig — RD cells were treated with CHX (100 μg/ml) for 4 h. Cell lysates were then separated by SDS-PAGE and western blotting was performed using VIMP and Herp antibodies. Herp expression served as a control molecule with a short half-life. (TIF) [file ppat.1006674.s007.tif]

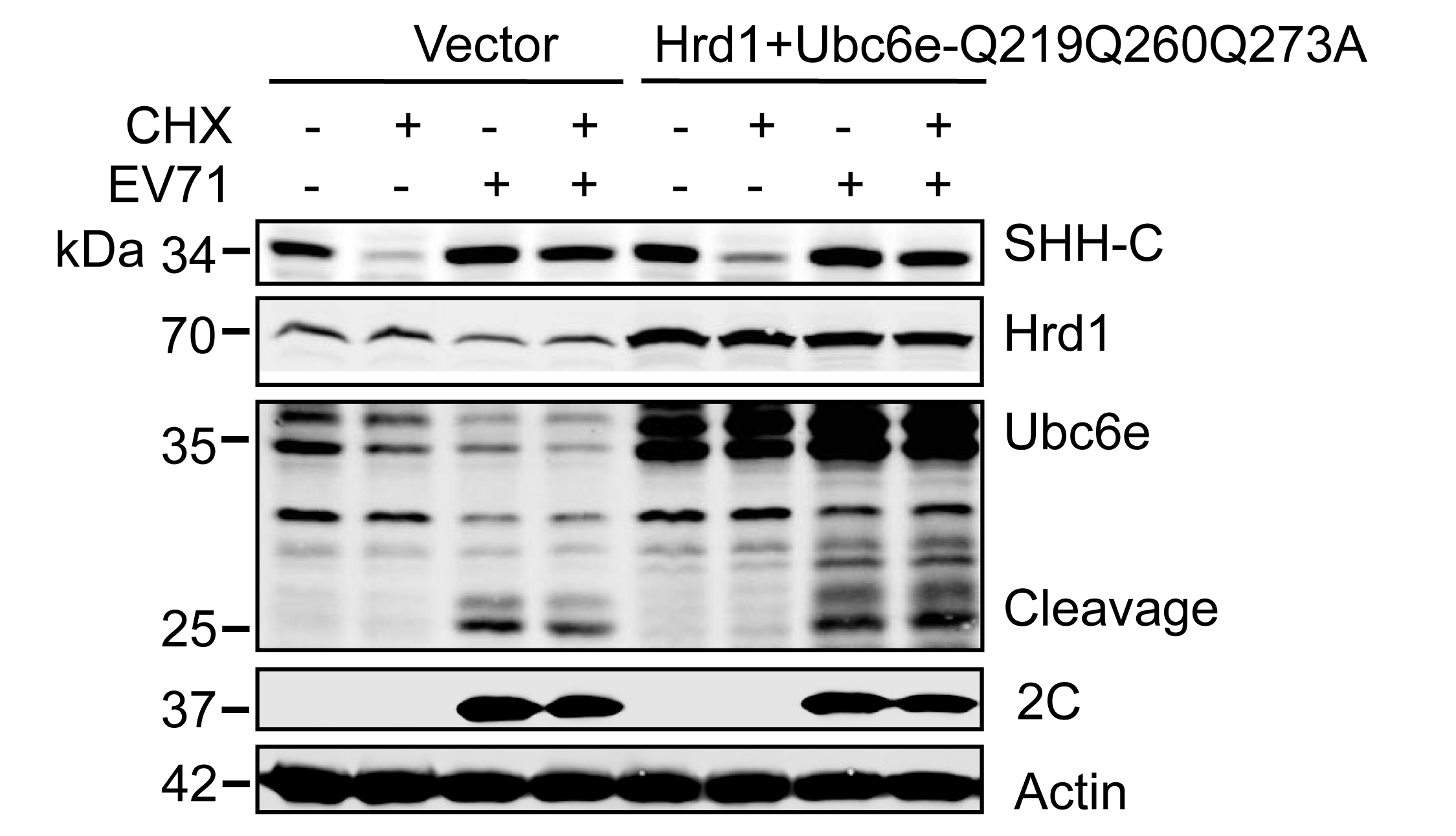

Supplement: S8 Fig — RD cells stably expressing SHH-FLAG were transfected with empty vector (control) or the Ubc6e triple-site mutant pVRC-Ubc6e-Q219Q260Q273A together with wild-type Hrd1. At 36 h post-transfection, mock infected (−) or infected (+) with EV71 (MOI = 10) for 12 h and then treated with (+) or without (−) CHX for 4 h. The cells were then harvested and the resulting cell lysates were analyzed by western blotting with the indicated antibodies. (TIF) [file ppat.1006674.s008.tif]

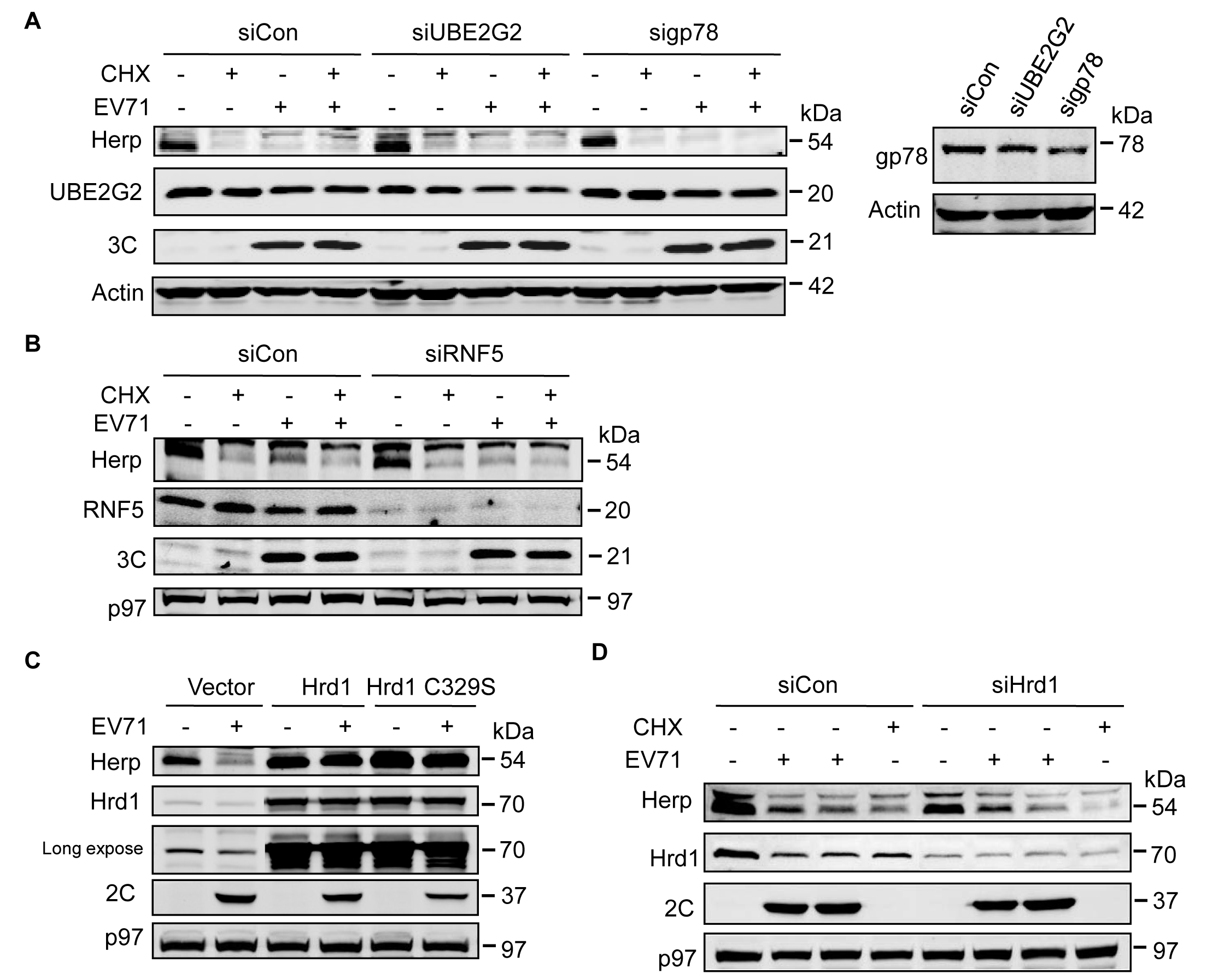

Supplement: S9 Fig — (A, B) RD cells were transfected with control and siRNA targeting UBE2G2 and gp78 (A), and RNF5 (B). At 36 h post-transfection, cells were mock-infected (−) or infected (+) with EV71 (MOI = 10) for 12 h and then treated with (+) or without (−) CHX for an additional 4 h. The cells were then harvested and cell lysates were analyzed by western blotting with the indicated antibodies. (C) RD cells were transfected with control or Hrd1 siRNA. At 36 h post-transfection, the cells were mock infected (−) or infected (+) with EV71 (MOI = 10) for 9 and 12 h, respectively. CHX-chased cells (100 μg/ml, 4 h) were used as a control for Herp downregulation. The cells were harvested and cell lysates were analyzed by western blotting with the indicated antibodies. (D) RD cells were transfected with empty vector, wide-type Hrd1, and Hrd1 C329S (dominant negative mutant). Thirty-six hours after transfection, cells were mock infected (−) or infected (+) with EV71 (MOI = 10) for 12 h. The cells were harvested and cell lysates were analyzed by western blotting with the indicated antibodies. (TIF) [file ppat.1006674.s009.tif]

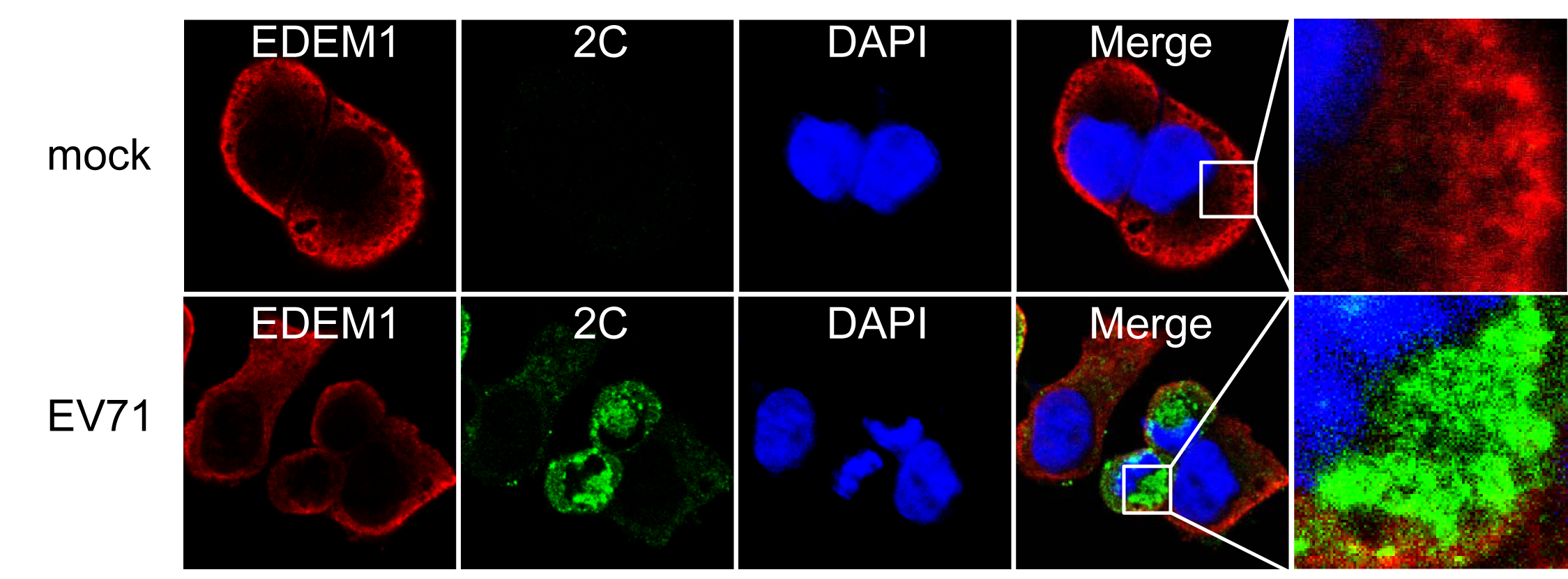

Supplement: S10 Fig — RD cells were mock-infected or infected with EV71 (MOI = 10) for 12 h. Then, immunostaining was performed to detect the intracellular distribution of EDEM1 and EV71 2C (EDEM1, red; 2C, green; nuclei, blue). The insets show magnified views of merged channels in the boxed region. (TIF) [file ppat.1006674.s010.tif]
