# Supplementary material for: Enterovirus 71 protease 2Apro and 3Cpro differentially inhibit the cellular endoplasmic reticulum-associated degradation (ERAD) pathway via distinct mechanisms, and enterovirus 71 hijacks ERAD component p97 to promote its replication
Source: PLoS Pathog. 2017 Oct 6;13(10):e1006674. doi: 10.1371/journal.ppat.1006674 (PMC5650186; doi:10.1371/journal.ppat.1006674)
Supplement: S1 Table — (DOCX) [file ppat.1006674.s011.docx]

**S1 Table. Sequences of siRNA duplexes used in this study**

| Protein | Target Sequence |
| --- | --- |
| EDEM1  (three mixture) | 5’-GCCGAAACCUCAUGAGUUATT-3’ |
|  | 5’-GCAUCACGUCAUUGACAAGTT-3’ |
|  | 5’-GAAUAAUAACUGACUCCAATT-3’ |
| OS9  (three mixture) | 5’-GGAAACACCUGCUUACCAATT-3’ |
|  | 5’-CAAGCCGACUCAAAGCAGUTT-3’ |
|  | 5’-GCAGCAUCGUCUUAAACGCTT-3’ |
| SEL1L  (three mixture) | 5’-GCUCAGUAGUACAGAGAAUTT-3’ |
|  | 5’-GGAUAUUCACCUUGCGAAATT-3’ |
|  | 5’-CGAGAGAGAUGUUUGAGAATT-3’ |
| Derl1 | 5’-UGGAUAUGCAGUUGCUGAUTT-3’ |
| Derl2  (three mixture) | 5’-CAAUCAACCUGGUGGAAUATT-3’ |
|  | 5’-CAAUAAUGCUCGUCUAUGUTT-3’ |
|  | 5’-CCAUCUAUUUUGAAAGCUATT-3’ |
| Hrd1  (three mixture) | 5’-CCAUGAGACAGUUCAAGAATT-3’ |
|  | 5’-GCAUGGCAGUCCUGUACAUTT-3’ |
|  | 5’-CAAGGUGUUCUUUGGGCAATT-3’ |
| gp78  (three mixture) | 5’-GCAAGGAUCGAUUUGAAUATT-3’ |
|  | 5’-CAAGUCUGCUGAUGAGAGATT-3’ |
|  | 5’-GCAGAAUGUCUCUUAAUAUTT-3’ |
| RNF5  (three mixture) | 5’-GCGACCUUCGAAUGUAAUATT-3’ |
|  | 5’-CGGCAAGAGUGUCCAGUAUTT-3’ |
|  | 5’-GAAGGUUGUCCCGCUUUAUTT-3’ |
| Ubc6e  (three mixture) | 5’-GCGCAGCCUUUAGAGGAUATT-3’ |
|  | 5’-GCCAUAGGUUCUCUAGAUUTT-3’ |
|  | 5’-CCAUAGGUUCUCUAGAUUATT-3’ |
| UBE2G2  (three mixture) | 5’-GUGAGAUGUUUCAUCCCAATT-3’ |
|  | 5’-AGAUGUUUCAUCCCAACAUTT-3’ |
|  | 5’-UGUUUCAUCCCAACAUCUATT-3’ |
| VIMP | 5’-ggaaccugauguuguuguuaa-3’ |
| UBXD8 | 5’-GAAGUUAUUUCACUAAUAATT-3’ |
| p97  (three mixture) | 5’-GAAUAGAGUUGUUCGGAAUTT-3’ |
|  | 5’-GGAGGUAGAUAUUGGAAUUTT-3’ |
|  | 5’-GGCCAAAGCCAUUGCUAAUTT-3’ |
| Npl4 | 5’-CAGCCUCCUCCAACAAAUCTT-3’ |
| Ufd1  (three mixture) | 5’-CAACUCAGCCGACUUAACATT-3’ |
|  | 5’-CUGUUCAAACUGACCAAUATT-3’ |
|  | 5’-GUGACAUGAACGUGGACUUTT-3’ |
| PI4KB | 5’-GGACUCACCAGCGCUCUAATT-3’ |
| GBF1 | 5’-CAACACACCUACUAUCUCUTT-3’ |
| ARF1  (three mixture) | 5’-GGUGGACAGCAAUGACAGATT-3’ |
|  | 5’-CCAUAGGCUUCAACGUGGATT-3’ |
|  | 5’-CGAUCCUCUACAAGCUUAATT-3’ |
