# Supplementary material for: Enterovirus 71 protease 2Apro and 3Cpro differentially inhibit the cellular endoplasmic reticulum-associated degradation (ERAD) pathway via distinct mechanisms, and enterovirus 71 hijacks ERAD component p97 to promote its replication
Source: PLoS Pathog. 2017 Oct 6;13(10):e1006674. doi: 10.1371/journal.ppat.1006674 (PMC5650186; doi:10.1371/journal.ppat.1006674)
Supplement: S1 Text — (DOCX) [file ppat.1006674.s012.docx]

# Supporting Materials and Methods

## Antibodies, reagents, and plasmids

PNGase F (P0704) was purchased from New England Biolabs. Annexin V-FITC/PI apoptosis assay kit (FAK011) was purchased from Neobioscience. pcDNA4-Hrd1 was purchased from Abgent, and pCDNA4-Hrd1 C329S were generated by site-directed mutagenesis. To construct pVRC-Ubc6e and pVRC-Ubc6e-Q219Q260Q273A, the cDNA was amplified by PCR from pCMV6-Ubc6e and pCMV6-Ubc6e-Q219Q260Q273A, respectively, and then cloned into the *Sal*I and *Xba*I sites of pVRC vector.

## Glycosidase digestion

RD cells stably expressing TTR D18G were collected and lysed in ice-cold lysis buffer. They were then incubated with PNGase F (1000 U) for 30 min at 37°C, separated by SDS-PAGE, and analyzed by western blotting.

## Flow cytometry

For analysis of apoptosis in EV71 infected RD cells, the cells were harvested, washed twice with PBS, incubated with FITC-labeled annexin V and stained with PI, and then analyzed by flow cytometry (BD CantoII). The annexin V-positive/PI-negative cells were considered to be apoptotic at the early period, annexin V-positive/PI-positive cells were considered to be apoptotic at the later period, whereas PI single positive cells were considered necrotic.
